# *Daphnia magna* Multigeneration Exposure to Carbendazim: Gene Transcription Responses

**DOI:** 10.3390/toxics11110918

**Published:** 2023-11-10

**Authors:** Ana Rita R. Silva, Patrícia V. Silva, Ana Raquel Soares, M. Nazaret González-Alcaraz, Cornelis A. M. van Gestel, Dick Roelofs, Gabriela Moura, Amadeu M. V. M. Soares, Susana Loureiro

**Affiliations:** 1 Centre for Environmental and Marine Studies (CESAM) & Department of Biology, University of Aveiro, 3810-193 Aveiro, Portugal; pverissimo@ua.pt (P.V.S.); asoares@ua.pt (A.M.V.M.S.); sloureiro@ua.pt (S.L.); 2 Department of Medical Sciences & Institute for Biomedicine (iBiMED), University of Aveiro, 3810-193 Aveiro, Portugal; ana.r.soares@ua.pt (A.R.S.); gmoura@ua.pt (G.M.); 3 Department of Agricultural Engineering of the E.T.S.I.A., Technical University of Cartagena, 30203 Cartagena, Spain; nazaret.gonzalez@upct.es; 4 Amsterdam Institute for Life and Environment (A-LIFE), Faculty of Science, Vrije Universiteit Amsterdam, De Boelelaan 1085, 1081 HV Amsterdam, The Netherlands; kees.van.gestel@vu.nl (C.A.M.v.G.); dick.roelofs@keygene.com (D.R.); 5 Keygene N.V., Agro Business Park 90, 6708 PW Wageningen, The Netherlands

**Keywords:** aquatic invertebrates, water pollution, pesticide, multigeneration testing, gene transcription, microarrays

## Abstract

The world population is experiencing colossal growth and thus demand for food, leading to an increase in the use of pesticides. Persistent pesticide contamination, such as carbendazim, remains a pressing environmental concern, with potentially long-term impacts on aquatic ecosystems. In the present study, *Daphnia magna* was exposed to carbendazim (5 µg L^−1^) for 12 generations, with the aim of assessing gene transcription alterations induced by carbendazim (using a *D. magna* custom microarray). The results showed that carbendazim caused changes in genes involved in the response to stress, DNA replication/repair, neurotransmission, ATP production, and lipid and carbohydrate metabolism at concentrations already found in the environment. These outcomes support the results of previous studies, in which carbendazim induced genotoxic effects and reproduction impairment (increasing the number of aborted eggs with the decreasing number of neonates produced). The exposure of daphnids to carbendazim did not cause a stable change in gene transcription between generations, with more genes being differentially expressed in the F0 generation than in the F12 generation. This could show some possible daphnid acclimation after 12 generations and is aligned with previous multigenerational studies where few ecotoxicological effects at the individual and populational levels and other subcellular level effects (e.g., biochemical biomarkers) were found.

## 1. Introduction

As the global population grows, the demand for increased agricultural production has become more pressing. Consequently, there has been a significant rise in the utilization of pesticides within agriculture practices in order to enhance crop yields and increase food production. Carbendazim (CBZ, methyl-2-benzimidazole carbamate) is an active ingredient in fungicides used to eradicate several pathogens. It has been applied for several years in agriculture for protecting crops (e.g., vegetables, fruits, and ornamental plants) and as a preservative in textiles, paints, and construction materials [1,2,3]. Although applied mostly to crops, carbendazim has been detected in surface waters in Thailand (Songkhla Lake Basin, Rattaphum catchment), Spain (Guadalquivir River Basin), and Chile (Traiguén River Basin) at concentrations of up to nearly 5 µg L^−1^ (measured in an agricultural–forestry basin), contributing to the contamination of the aquatic environment and possibly posing a risk to non-target organisms, such as aquatic invertebrates [4,5,6]. Carbendazim has been detected in different water matrices with concentrations ranging from 0.003 µg L^−1^ to 5 µg L^−1^. In detail, carbendazim has been detected in effluents of wastewater treatment plants in different European countries and in one of the major European rivers, the Rhine River, with it being detected from the Swiss Alps through Switzerland, France, Germany, and the Netherlands to the North Sea; in Swedish agricultural areas, it was detected in stream samples; and it has also been detected in Brazil (São Paulo River) and China (groundwater) [6,7,8,9,10,11,12,13]. In a previous report, the maximum permissible concentration (MPC) for carbendazim in water was found to be 0.6 µg L^−1^; this value was calculated using a species sensitivity distribution curve with a representative community (with eight taxonomic groups) [14]. The environmental release of carbendazim might be continuous, as its application is not restricted to one season. In 2020, carbendazim was prohibited for all application types in the European Union due to its toxicity, including embryonic, reproductive, developmental, and hematological toxicity described in different organisms (e.g., for fish and mice) [15,16]. Moreover, carbendazim is considered an anthropogenic contaminant of the highest concern and is prioritized based on its risk score (the sum of frequency of appearance, frequency of exceedance, and extent of exceedance) [8,11]. Considering all previously mentioned issues, carbendazim should be studied holistically, understanding its effect from the gene transcription level to the population level, under long-term exposure, with this being highly relevant for assessing the environmental risk of this compound in freshwater ecosystems. This is especially relevant considering that this controversial compound, carbendazim, was reintroduced onto the market in Europe in 2021 (approved by the European Commission for use in film and other construction material preservatives under the biocidal products Regulation (BPR)—Regulation (EU) 2021/348) and is still widely used and found nowadays in different compartments throughout the world [15]. 

Cladocerans are one example of aquatic organisms inhabiting surface waters that may be affected by carbendazim. The sensitivity of the cladoceran *Daphnia magna* to different compounds has already been demonstrated. In addition, *D. magna* is a standard test species and one of the most studied organisms in aquatic ecotoxicology [17]. Until recently, ecotoxicity testing has been mainly focused on standard endpoints (e.g., immobilization or reproduction) to assess chemical effects. However, effects at the subcellular level (e.g., gene transcription) considering long-term exposure to environmentally measured concentrations have received less attention. Genomic tools, such as gene expression microarrays and RNASeq, can be used to understand the molecular mechanisms of toxicity in organisms [18]. When this multigenerational test was first performed, microarray analysis was the most advantageous technique available, as the genome of *D. magna* was not assembled at that time, which hindered RNASeq analysis. We acknowledge that the technique of RNASeq may currently provide advantages over the microarray as the *D. magna* genome has been assembled since 2019 (REF—PMID 30826642). However, while RNA-Seq holds considerable promise as a technology, its potential advantages over microarrays in toxicogenomic studies remain somewhat elusive. The published research has yet to comprehensively assess the concordance/discordance in differentially expressed genes between these two technologies in the context of toxicological investigations. Several microarrays have been developed for *D. magna* to assess transcriptomic changes and test the effects of chemical compounds [19,20]. The closely related species *Daphnia pulex* was the first crustacean to have its genome sequenced, and this information has helped in identifying genes of interest in *D. magna* by homology [21]. 

Contaminants might persist in time in aquatic environments, leading to the accumulation of epigenetic marks and, therefore, possible phenotype changes in subsequent generations [18]. As recently highlighted by Zhou and their colleagues (2023) [15], the long-term effect of carbendazim should be considered as carbendazim might enter the human body via the food chain (e.g., via fish) and skin contact. Gene transcription endpoints may serve as valuable early biomarkers of effects caused by environmental stressors. To our knowledge, no information exists on how the *D. magna* transcriptome can be affected when exposed for long periods to carbendazim.

Considering the above, the present study aimed to assess the effects of long-term exposure to carbendazim (5 µg L^−1^) on *D. magna* at the gene transcription level using a multigenerational approach with twelve generations (F0-F12). The selection of the carbendazim concentration considered the concentrations found in surface waters, with a particular focus on the upper limit of the range [6] and previous toxicity tests [22,23,24]. Multigenerational effects of carbendazim in *D. magna*-specific subcellular (DNA damage and biochemical biomarkers) and individual/populational level endpoints (such as longevity/reproduction) were previously evaluated by us [24]. Here, we complement that previous study with a multigenerational gene expression analysis of the effects of carbendazim exposure to answer the following questions: (i) does carbendazim induce changes in the *D. magna* transcriptome? (ii) Are transcriptome-induced changes related to the previous subcellular- and individual-level effects observed? (iii) How do these changes vary in time with continuous exposure? 

## 2. Materials and Methods

### 2.1. Test Organism

*Daphnia magna* Straus clone K6 (originally from the Laboratory for Ecophysiology, Biochemistry and Toxicology, Department of Biology, University of Antwerp, Belgium) was obtained from continuous cultures and maintained under laboratory conditions at the University of Aveiro (Aveiro, Portugal). The test organisms were cultured in the American Society for Testing and Materials (ASTM) moderated-hard-water medium [25] at a temperature of 20 ± 1 °C and for a 16 h light–8 h dark photoperiod. The daphnid culture medium was renewed three times a week, and during each renewal, the organisms were fed with *Raphidocelis subcapitata* at a concentration of 3 × 10^5^ cells mL^−1^ and supplemented with an organic extract (Marinure seaweed extract, supplied by Glenside Organics Ltd., Scotland, UK). 

### 2.2. Test Chemical

Carbendazim stock solutions (CAS No. 10605-21-7, 99.4% purity, Bayer, Monheim am Rhein, Germany) were prepared in ASTM medium and used for the exposure media. Chemical analyses were performed at Marchwood Scientific Services (Southampton, UK) for carbendazim concentration verification. The analyses for carbendazim were performed by liquid chromatography-mass spectrometry (LCMS-MS) using the QuERCHERS method. Approximately 200–300 mL of the sample was extracted with 20 mL of acetonitrile (containing 1% acetic acid), followed by a partitioning step with magnesium sulfate and a subsequent buffering step with sodium acetate. An aliquot was mixed with methanol, and the extract was injected directly into the LCMS-MS system (instrument Agilent 6410 Triple Quad LCMs-MS) without any clean-up. A 10 µL injection volume was used. Standards were prepared in solvents at seven levels with recoveries ranging between 70% and 120%. The degradation rate constant (*K*_0_) was calculated to determine chemical decay in time by the following equation (1):

*C_t_* = *C*_0_*e*^−*K*^_0_^*t*^ (1)

where *C*_0_ is the initial external concentration (µg L^−1^), *K*_0_ is the degradation rate constant of the chemical in the medium (hour^−1^) and *t* is time (in hours) [26].

### 2.3. Multigenerational Experimental Setup

Preliminary toxicity (immobilization, feeding inhibition, and reproduction) tests were conducted to determine the toxicity of carbendazim to *D. magna* (see Silva et al., 2015 [22]). The no observed effect concentration (NOEC) value obtained for the reproduction test (i.e., number of neonates; [17]), corresponding to 5 µg L^−1^ [22], was selected as the exposure concentration for daphnids throughout twelve generations. The selected concentration allows for the feasibility of conducting a multigenerational experiment maintaining a stable population (i.e., avoiding mortality) to enable sampling and effect analysis. Moreover, carbendazim has been found in European water bodies in concentrations reaching ≈ 5 µg L^−1^ [6], showing the environmental relevance of this concentration. The experimental design is described in detail in Figure S1. 

For the multigenerational experiment, three replicate glass vessels containing 1 L of culture medium and 20 daphnids were prepared with either clean medium (control) or medium spiked with carbendazim (5 µg L^−1^). In this work, the designation “clean medium” is used instead of control to be consistent with our previous studies [23,24]. The multigenerational test was based on the OECD 211 guideline with several adaptations, namely the number of replicates and the number of organisms per replicate [17]. In detail, each replicate consisted of ASTM medium with *R. subcapitata* (concentration of 3 × 10^5^ cells mL^−1^) and supplemented with an organic extract (Marinure seaweed extract, supplied by Glenside Organics Ltd, Scotland, United Kingdom); the medium was renewed three times a week (Monday, Wednesday, and Friday). Neonates from the third brood of the F0 generation were used to start the next generation (F1) under the same conditions; then, the third brood neonates from the F1 generations were used to start the next generation (F2), and the same procedure was applied to all generations until the F12 generation was reached (either in clean medium or in carbendazim), therefore constituting a continuous exposure trial.

### 2.4. Gene Transcription Analysis

#### 2.4.1. RNA Extraction

For the microarray analysis, neonates at less than 24 h old were sampled from the F0 and F12 generations from both clean medium and carbendazim exposure. The details for the experimental design are in Figure S1. Three replicates per treatment were used, with each replicate consisting of 5 daphnids maintained under the same conditions and pooled together—daphnids in clean medium maintained in clean medium and daphnids in carbendazim maintained in carbendazim (5 µg L^−1^) until reaching 10 days of age. This age (10 d) allowed the 5 daphnids (per replicate) to have enough genetic material to proceed with RNA extraction. The organisms were then collected, shock-frozen in liquid nitrogen, and stored at −80 °C until RNA extraction.

RNA was extracted using the Trizol^®^ method(Invitrogen, Waltham, USA), followed by a column purification step using the RNAeasy kit^®^ Qiagen(Hilden, Germany), and stored at −80 °C. Prior to storage, the purity of the RNA samples (A260/280 and A260/230 ratios) was determined using a Nanodrop^®^ 2000c spectrophotometer (Nanodrop Technologies, Wilmington, USA) and checked for integrity in an Agilent 2100 Bioanalyzer (Agilent Technologies, Santa Clara, CA, USA). 

#### 2.4.2. Gene Expression mRNA Microarrays

Following RNA extraction, the samples were prepared for labelling and hybridization on the microarray. The custom *D. magna* microarray used in the present work was designed by EcoArray by Agilent Technologies (8 individual arrays per slide) and was composed of 7370 probes. Each biological replicate was individually hybridized on the array. A single-color approach was applied, using the Agilent one-color RNA Spike-In Kit (Agilent Technologies), following the manufacturer’s protocol. The samples were hybridized for 17 h at 65 °C with a rotation of 10 rpm; then, the microarrays were washed using the Agilent Gene Expression Wash Buffer Kit (Agilent Technologies) according to the manufacturer’s protocol. 

#### 2.4.3. Microarray Data Extraction and Analysis

Agilent DNA microarray scanner G2505B (Agilent Technologies) was used for scanning. The array did not contain the complete genome of *D. magna*; consequently, some genes were missed, and it was impossible to detect the regulation of non-coding genes (limitation of microarrays). Probe signal values were extracted from microarray scan data using Agilent Feature Extraction Software 12.0. The data were median normalized using BrB-Array tools v4.4.1 software(The EMMES Corporation, Rockville, Maryland). Differentially expressed genes in the F0 and F12 generations were identified using Multiple Experiment Viewer (TMev) software v4.9. An unpaired t-test with standard Bonferroni correction was performed to identify genes that demonstrated statistically (*p* < 0.05) significant differences. First, differences between F0 and F12 daphnids in clean medium and between F0 and F12 daphnids in carbendazim-spiked medium were assessed to analyze differential or similar transcription profiles between generations. Genes that significantly varied in transcription at F0 vs. F12 in clean medium were removed from the list of differentially transcribed genes of F0 vs. F12 with carbendazim to distinguish the effects of carbendazim. Differences in gene transcription between the clean medium and carbendazim-exposed daphnids for both F0 and F12 generations (clean medium vs. carbendazim in F0 and clean medium vs. carbendazim in F12) were also evaluated. Genes were considered up-regulated when the fold change was higher than 1.5 and down-regulated when the fold change was lower than −1.5. Blast2GO (Biobam Bioinformatics Support, Valencia, Spain) was used to annotate the sequences [27]. The PANTHER tool was used to identify GO terms for the deregulated genes (www.pantherdb.org/, accessed on August 2023).

#### 2.4.4. Microarray Data Submission

The microarray raw data have been submitted to the Gene Expression Omnibus database and given the following accession number: GSE78120.

## 3. Results and Discussion

### 3.1. Chemical Analyses

The results of the chemical analyses carried out within the 48 h period of media renewal showed that the carbendazim concentration decreased over time, with a decay rate (*K_0_*) of 0.03 h^−1^ (st. error = 0.005) (this is also described in Silva et al. (2015) [22]), corresponding with a half-life of 23 h. Although carbendazim is degraded in ASTM medium, the presence of different metabolites cannot be disregarded. One already identified major metabolite of carbendazim is 2-aminobenzimidazole [28,29]. 

### 3.2. Multigenerational Responses

Multigenerational approaches or long-term exposure are not often used to depict effects at the transcriptome level. Although this subcellular endpoint may be considered an early effect biomarker, we intended to further understand the time dependency of gene transcription in response to carbendazim. On one side, after short-term exposure, the immediate responses of microcrustaceans to carbendazim might exhibit rapid changes in gene transcription. These immediate responses can involve activating or suppressing specific genes involved in stress responses, detoxification, and repair mechanisms. On the other hand, prolonged exposure to carbendazim might lead to chronic responses in gene transcription which can result in toxic effects at the individual or population level or lead to possible acclimation [30]. These long-term changes can involve the activation of genes related to adaptive responses, energy allocation, and, potentially, the development of tolerance. Thus, in the present study, the molecular impact of carbendazim on *D. magna* was investigated under a multigenerational approach, and gene transcription results were linked, when possible, to effects at the subcellular (e.g., biochemical alterations) and individual levels that were observed in previous experiments [22,23,24]. 

**Figure 1:**
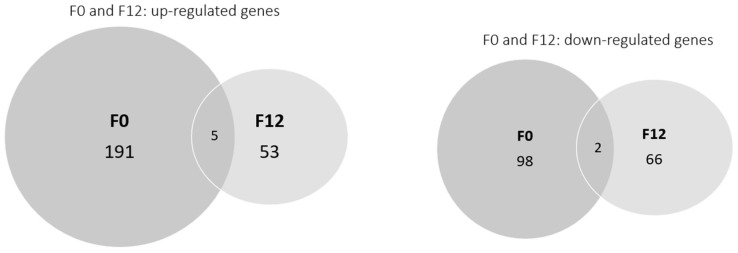
Overview of genes in response to carbendazim in the *Daphnia magna* multigenerational experiment. Venn diagram showing the overlap of significantly up- and down-regulated genes in both F0 and F12 generations.

The list of up- and down-regulated genes for F0 vs. F12 in clean medium and F0 vs. F12 in carbendazim is presented as Supplementary Data (Table S1). Differentially transcribed genes decreased from generation F0 to F12 for daphnids exposed to carbendazim (Figure 1; Table S2). Few differentially expressed genes were common between the F0 and F12 generations, with them sharing only five up-regulated genes and two down-regulated genes (Table 1).

Variability in gene transcription in daphnids in controls (clean medium) from different generations was found in the present work and has been reported in other studies [18,31]. This difference might be attributed to differences in physiological processes, for instance, in reproductive cycles or molting phases [18,31]. Some of the described differential transcription of genes may result from fluctuations in homeostasis during culturing of these animals [32]. Gene transcription variability might provide some insights regarding the changes in responses (in LC_50_ or EC_50_ values) in daphnids kept under the same conditions (e.g., medium, temperature, light:dark photoperiod, and type of food) and exposed to the same compound in different times. 

The complete list of up- and down-regulated genes for generations F0 (clean medium vs. carbendazim) and F12 (clean medium vs. carbendazim) is presented in the Supplementary Data (Table S2). In the microarray experiment, the hierarchical clustering showed a division between samples from the clean medium (Control) and samples from carbendazim exposure (CBZ) for both F0 and F12 generations (Figure S2).

#### 3.2.1. Gene Transcription in the F0 Generation (Clean Medium versus Carbendazim)

**Figure 2:**
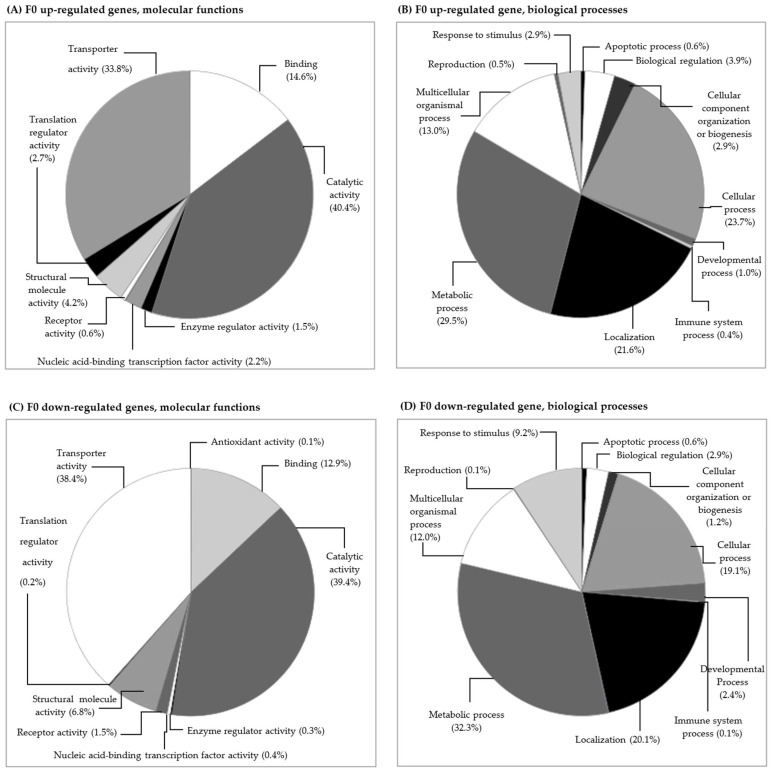
Functional category classification of differentially expressed genes of *Daphnia magna* exposed to 5 µg L^−1^ carbendazim: (**A**) F0 generation, up-regulated genes and molecular functions, (**B**) F0 generation, up-regulated genes and biological processes, (**C**) F0 generation, down-regulated genes and molecular functions, and (**D**) F0 generation, down-regulated genes and biological processes. The percentage of affected genes is presented between brackets.

The analysis between clean medium and carbendazim-exposed daphnids resulted in 191 up-regulated and 98 down-regulated genes (Figure 1, Table S2). The functional categories of these genes were identified using the PANTHER tool and are shown in Figure 2. In the F0 generation, carbendazim caused changes in genes involved in molecular functions such as catalytic activity, transporter activity, and binding (Figure 2A,C). These changes suggest that *D. magna* exposed to carbendazim experienced molecular and physiological alterations. The most affected categories for biological processes were metabolic processes, cellular processes, localization, and multicellular organismal processes (Figure 2B,C). These changes can have various consequences, from metabolic disruptions and adaptive responses to potential long-term effects on populations and ecosystems.

##### Gene Transcription and Its Relation to Different Subcellular Endpoints

Several studies have already demonstrated the genotoxic potential of carbendazim for different species [22,33,34]. Carbendazim did affect DNA integrity in different invertebrate species, from the marine invertebrate *Donax faba* to the freshwater microcrustacean *D. magna* [22,33]. Our previous experiments showed that DNA damage was one of the most significant effects in *D. magna* upon exposure to carbendazim. In that study, *D. magna* (<24 h neonates) showed an increase in DNA damage, as DNA strand breaks evaluated using the comet assay, after 24 h of exposure to carbendazim at concentrations of 5, 20, and 25 µg L^−1^ [22]. In the present study, in the F0 generation, a 6.98-fold transcriptional induction of a homologous gene coding for a ubiquitin-conjugating enzyme E2 variant 2-like protein (UBE2V2, *Apis mellifera*) and 4.44-fold for an exonuclease ABC, subunit A (uvrA, *Halothermothrix orenii*) occurred (Table S2). Ubiquitin is known to regulate various cellular processes, including DNA damage response and repair and exonuclease ABC, subunit A is a gene that encodes a protein involved in DNA repair processes [35,36]. In fact, the gene encoding for histone deacetylase Rpd3 (HDAC Rpd3) (*Tribolium castaneum*) was up-regulated 14.83-fold. HDAC plays an important role in the regulation of gene expression, cell cycle, and apoptosis and is responsible for the removal of acetyl groups, increasing ionic interactions between the positively charged histones and negatively charged DNA, which produces a more compact chromatin structure and represses gene transcription and is therefore associated with gene silencing [37]. These histone deacetylases are required to activate DNA damage-inducible genes [38]. The coding gene for DNA polymerase epsilon catalytic subunit A (*Plasmodium falciparum*), which plays a role in DNA repair and chromosomal DNA replication [39], was −2.21-fold down-regulated, as well as a gene encoding for a DNA mismatch repair protein (Xanthomonas campestris) (−2.63-fold). Novais et al. (2012) [40] also found that DNA damage and repair processes were significantly affected after only 2 days of exposure of *Enchytraeus albidus* to carbendazim. In their study, genes coding for intermediate filament proteins involved in DNA repair were significantly induced at all concentrations, supporting the genotoxic effects of carbendazim. Thus, DNA damage effects were related to some of the presented genes, suggesting that gene transcription may be in fact an early warning signal of exposure to carbendazim, even at low concentrations of the compound.

In addition, the heat-shock protein 70 (HSP70) (*Entamoeba histolytica*) was transcriptionally repressed (−3.47-fold). This protein is important as an ATP-dependent molecular chaperone, helping in polypeptide folding and the targeting of proteins for lysosomal degradation [41]. Other roles of HSP 70 include responses to cellular stress [42] and the blocking of apoptosis through the binding of a protease activating factor-1, preventing the constitution of the apoptosome [42]. Down-regulation of HSP70 probably slows/hampers cellular recovery [41]. In the context of the present study, transcriptional repression of HSP70 might indicate that the cellular mechanisms for repairing damage or counteracting the effects of carbendazim are compromised, which may help explain the DNA damage observed in *D. magna* in our previous studies [22,24].

In a similar multigenerational experiment, GST activity increased in F0 carbendazim-exposed daphnids (also at 5 µg L^−1^) compared with the F0 clean medium [24]. In the present study, the gene coding for a papilin-like protein (*Apis mellifera*) was 3.41-fold up-regulated. This gene is considered to encode a serine-type endopeptidase inhibitor, preventing, or reducing the activity of serine-type endopeptidases. A 5.02-fold up-regulation of glutathione S-transferase T1 (GSTT1) (*Apis mellifera*) homologues was observed. GST plays an important role in the biotransformation/cellular detoxification processes of various chemicals and defense against peroxidative products of DNA [43,44]. These results suggest that the transcriptional up-regulation of genes related to anti-oxidant activities indicates a possible response to oxidative stress [45], and thus, it may be related to the biomarker effects observed by Silva et al. (2019) [24]. 

The gene coding for a protein homologous to Gaba(A) receptor-associated protein (*Branchiostoma belcheri* tsingtaunese) was −2.83-fold down-regulated; this protein mediates inhibitory neurotransmission [46]. Likewise, there was a 9.41-fold up-regulation of the gene encoding slowmo (*Bombyx mori*), known to cause inhibition of neurotransmitter release [47]. The gene coding for glucosamine 6-phosphate N-acetyltransferase (GNPNAT1) (*Anopheles gambiae*) that participates in glutamate metabolism was −3.75-fold repressed. In the study of Vandegehuchte et al. (2010b) [41], the GNPNAT1 gene was up-regulated in *D. magna* after zinc exposure (F0). Glutamate is an excitatory neurotransmitter that plays a principal role in neural activation [48], which could suggest that carbendazim might cause adverse neural effects on *D. magna*. However, there are no results of previous experiments with carbendazim to support this hypothesis. 

Several transcripts involved in the electron transport chain system and oxidative phosphorylation were affected: up-regulation of genes encoding for ATP synthase, cytochrome b (cytb) (5.76-fold), cytochrome c1 (cytc1) (7.97-fold), cytochrome oxidase subunit II (COII) (5.71-fold), and cytochrome c oxidase (cox) (4.55-fold) and down-regulation of NADH dehydrogenase I (−20,664.18-fold) and cytochrome b-c1 complex (cyt b/c1) subunit 2 (−3.79-fold). These results suggest that carbendazim might affect mitochondrial activity and consequently ATP production, as already observed after *E. albidus* exposure to carbendazim [40]. *D. magna* showed induction of ATP synthase when exposed to the pesticide propiconazole for 4 days, and the authors suggested that it might be related to increased demands of energy (such as ATP) to cope with stress/detoxification [19]. It might be suggested that *D. magna* could be allocating energy to cope with stress/detoxification caused by carbendazim exposure. Therefore, less energy was available for reproduction, with a consequent decrease in the number of neonates, as observed in the study of Silva et al. (2015) [22]. Related to energy sources, carbohydrate metabolism was also affected, with genes also being up-regulated: polypeptide N-acetylgalactosaminyltransferase 5 (GALNT5) (18.77-fold), UDP-glucosyl transferase family protein (13.07-fold), and multifunctional fatty acid oxidation complex subunit alpha (fadJ) (6.24-fold).

##### Gene Transcription and Its Relation to Individual/Populational Endpoints

Homologous genes for proteins related to several metabolic pathways were affected as well. In the F0 generation, up and down transcription of genes involved in lipid metabolism were observed: up-regulation of glycerol-3-phosphate dehydrogenase (GAPDH) (9.74-fold), hydroxysteroid dehydrogenase-like 2 (hsdl2) (12.00-fold), and fatty acid desaturase (FADS) (8.57-fold); down-regulation of hydroxyacyl dehydrogenase subunit A isoform 3 (−2.91-fold), pyrroloquinoline-quinone aldehyde dehydrogenase (−3.49-fold) and MCEE. Lipids are known to be specifically involved in egg production in cladocerans [49]. The transcriptional deregulation of genes involved in lipid metabolism may be one of the factors influencing the reduction in the number of neonates of F0 carbendazim-exposed *D. magna* when compared with a clean medium [22,24,50]. 

Two genes involved in embryogenesis were affected [51]: a homologous gene coding for the oocyte maturation factor Mos (*Anas poecilorhyncha*) was 7.30-fold up-regulated and a homologous MAST1 gene (*Rattus norvegicus*) was −3.03-fold repressed. Exposure to carbendazim has been shown to interfere with microtubule assembly, causing a gradual disappearance of microtubules [52]. Novais et al. (2012) [40] observed down-regulation of the encoding gene for Stathmin 1 oncoprotein 18, which is directly involved in microtubule assembly/disassembly [53]. In several studies, carbendazim also caused a concentration-related decrease in the number of *D. magna* neonates and increased the number of aborted eggs, which is possibly related to mitosis inhibition during egg division in the brood pouch [22,50,54]. 

#### 3.2.2. Gene Transcription in the F12 Generation (Clean Medium versus Carbendazim)

**Figure 3:**
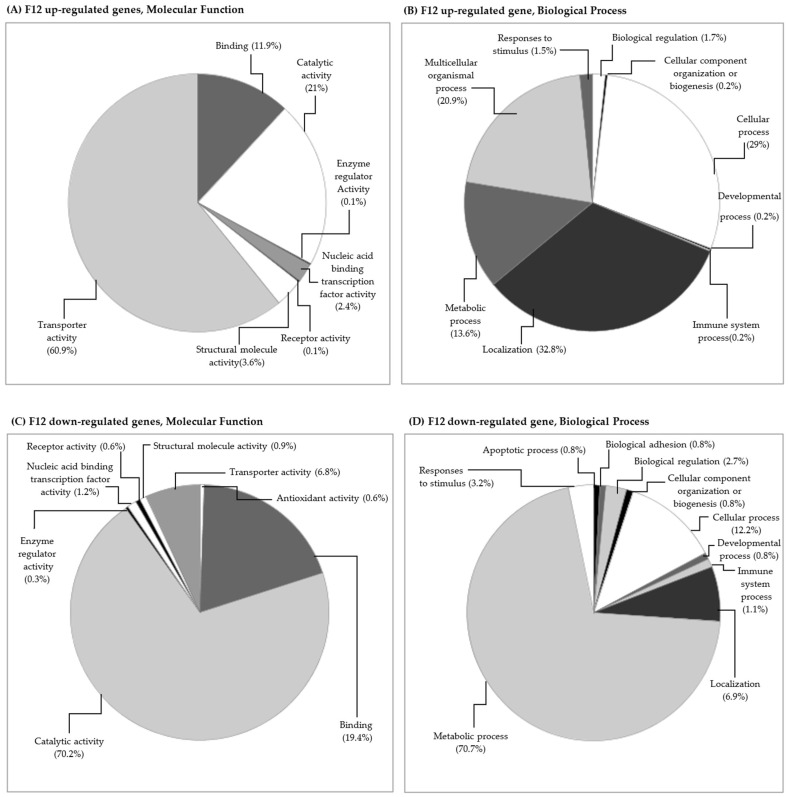
Functional category classification of differentially expressed genes of *Daphnia magna* exposed to 5 µg L^−1^ carbendazim: (**A**) F12 generation, up-regulated genes, and molecular functions, (**B**) F12 generation, up-regulated genes and biological processes, (**C**) F12 generation, down-regulated genes and molecular functions, and (**D**) F12 generation, down-regulated genes and biological processes. The percentage of affected genes is presented between brackets.

A lower number of differentially transcribed genes was observed for the F12 generation compared with the F0 generation: 53 genes were up-regulated and 66 were down-regulated (Figure 1, Table S2). In the F12 generation, the up-regulated genes were reduced almost four times, and the down-regulated genes were reduced almost two times when compared with the F0 generation. Vandegehuchte et al. (2010c) [55] also found a decrease in differentially expressed transcripts throughout three generations of *D. magna* exposed to zinc (388 µg L^−1^). Regarding individual endpoints, zinc exposure negatively affected body length and reproduction in the second generation compared to the control [55]. However, no differences were observed between treatments and the control in the third generation, suggesting that the organisms could have acclimated to zinc [55]. Similarly, in the F12 generation of daphnids exposed to carbendazim, a lower number of processes were affected along with a decrease in the number of up and down-regulated genes compared to the F0 generation. In the F12 generation, some of the categories were similar to the F0 generation. After 12 generations of daphnids exposed to carbendazim, changes in the gene transcription of proteins involved in some molecular functions, causing changes mainly in transporter, catalytic, and binding activity, were found (Figure 3A,C). The biological processes with the most affected genes were localization and metabolic, cellular, and multicellular organismal processes (Figure 3B,D).

##### Gene Transcription and Its Relation to Different Subcellular Endpoints

In the F12 generation, genes involved in ATP production were also affected, namely the NADH dehydrogenase subunit 6 (ND6) (*Mustelus manazo*), which was 15,117.52-fold up-regulated, and cytb, which was found to be −6.30-fold repressed. cytb is involved in ATP production as it is a crucial component of the mitochondrial electron transport chain, which plays a central role in oxidative phosphorylation. Disruptions in cytb function could have wide-ranging consequences for cellular energy metabolism and organismal performance.

Similar to what occurred in the F0 generation, some genes involved in neurotransmission processes were deregulated. The gene coding for the homologue protein testican-1-like in *Apis mellifera*, a protein involved in several neural mechanisms in the central nervous system [56], was also 1.95-fold induced (Table S2). 

The double-stranded RNA-activated protein kinase 1 (PKR1) (*Tetraodon nigroviridis*), responsible for apoptosis induction [57], was 35,149.50-fold up-regulated. The fem-1 homolog B (*Tribolium castaneum*) was −8.43-fold repressed. This gene encodes a protein that belongs to the death receptor-associated family of proteins and is therefore also implicated in apoptosis [58]. This protein is also involved in regulating DNA damage checkpoints, and consequently, its repression might have consequences for DNA damage. Jiang et al. (2014) [59] studied the effects of carbendazim (after 4 days of exposure to 4 µg L^−1^ of carbendazim) in the zebrafish *D. rerio* and identified differential transcription of genes playing a critical role in cell apoptosis pathways. Considering that some genes related to apoptosis were affected upon carbendazim exposure, further investigations should be performed to understand how these genes influence the apoptosis process in *D. magna*. In cell lines and mammalian tissues, carbendazim was shown to induce toxicity, mainly driven by oxidative stress which leads to apoptosis [38].

Furthermore, in the F12 generation, deoxyuridine triphosphatase (dut) (*Homo sapiens*), an enzyme of nucleotide metabolism also involved in DNA replication, was 3.21-fold up-regulated. Curtin et al. (1991) [60] reported that an increase in intracellular dut in human lung carcinoma A549 cells might lead to DNA strand breaks and consequently cell death. This might be related to the DNA strand breaks in *D. magna* exposed to carbendazim in our previous experiments [22,23,24]. In addition to this, the common up-regulated genes between F0 and F12 generations included a gene similar to dispatched homolog 1 (Disp1) and a gene similar to the YY1 transcription factor (Table 1). Disp1 is related to organ morphogenesis and hedgehog receptor activity, which is important for proper development in embryonic cells [61]. The YY1 transcription factor has an important role in biological processes, including embryogenesis, replication, and cellular proliferation, with it having important properties that initiate suitable cellular development [62]. YY1 might activate the p53 tumor-suppressor protein in response to genotoxic stress [63]. 

Previous studies have shown that the biochemical biomarkers cholinesterase, catalase and Glutathione-*S*-transferase and Lipid peroxidation tended to be similar after 12 generations of exposure to carbendazim (when compared with daphnids always in clean medium) [24]. This convergence pattern was also observed in assessments of energy availability and consumption [24]. However, DNA damage emerged as the most sensitive and consistent subcellular parameter in *D. magna* exposed to carbendazim, with it exhibiting a progressive increase across generations [23] without evident associated consequences for other endpoints.

##### Gene Transcription and Its Relation to Individual/Populational Endpoints

Genes involved in embryonic development were differentially expressed, with (18,679.08-fold) up-regulation of the gene coding for a protein with homology to Pax-6 protein in *Euprymna scolopes* [64] and the repression (−5.79-fold) of the gene encoding for the protein bicaudal C (*Tribolium castaneum*) [65]. Carbendazim acts on cell division, inhibiting the development of the germ tubes in the nucleus [22,51,53], which could be related to the decrease in the reproduction capacity in *D. magna* observed in our previous studies [22,51].

The present study contributed to unravelling alterations at the gene transcription level in daphnids exposed to an environmentally measured concentration of carbendazim. Long-term exposure to carbendazim at low concentrations (5 µg L^−1^) showed few effects at the individual and population levels throughout the generations (until F12) [24]. Individual effects were mainly observed when assessing daphnids’ longevity, which slightly decreased in F12 carbendazim-exposed daphnids compared to daphnids in (F12) clean medium [24].

Notwithstanding this, the deregulated genes obtained at 5 µg L^−1^ demonstrated in the present study are related to effects at the individual and population levels under exposure to higher carbendazim concentrations. The lowest concentrations affecting neonate production or aborted eggs at the population level were 20 and 35 µg L^−1^, respectively [22]. 

Changes in mRNA levels are good indicators of gene expression, though they cannot be used alone to directly correlate with protein expression due to varied post-transcriptional mechanisms involved in synthesizing the native protein. Additional proteomics and metabolomics studies must be conducted to determine the expression of a protein encoded by a deregulated gene. Although we cannot extrapolate the mechanism of action of carbendazim solely based on transcriptomics data, we have gathered valuable information on the influence of carbendazim at the gene transcription level that might be used as biomarkers of exposure. Considering the time-dependency of gene transcription responses in the present multigenerational experiment with *D. magna*, these data are crucial for a comprehensive understanding of the biological, ecological, and regulatory implications.

Changes in gene transcription patterns observed from the F0 to F12 generation of carbendazim exposure suggest that *D. magna* is undergoing acclimation to the pesticide. However, the specific mechanisms and the extent of acclimation are complex and may involve, for instance, trade-offs in resource/energy allocation. Further research is needed to unravel the full extent of acclimation and its consequences in this context. 

## 4. Conclusions

In the present study, we show that exposure to an environmentally measured concentration of carbendazim caused changes at the gene transcription level in F0 and F12 generations of *D. magna*. Answering the questions initially raised, it may be concluded that:

(i) These transcriptional changes were mostly in genes involved in the response to stress, DNA replication/repair, neurotransmission, protein biosynthesis, ATP production, and lipid and carbohydrate metabolism.
(ii) The transcriptome results from the present study seem to support the genotoxic effects of carbendazim previously described using the comet assay and the increase in the number of aborted eggs with the decreasing number of neonates produced [22,24].
(iii) These changes were not maintained over time, since a lower number of differentially transcribed genes was observed after twelve generations of daphnids exposed to carbendazim, and the pathways were differentially affected in the F0 and F12 generations, possibly showing some acclimation.

## Figures and Tables

**Table 1:** Gene ID and description of common a) up-regulated and b) down-regulated genes in the F0 and F12 generations of *Daphnia magna* following exposure to carbendazim.

	Gene ID	Gene Description [Species]
Up-regulated genes	YP_548045	hypothetical protein Bpro_1196 [*Polaromonas* sp.]
	AAY54998	IP06749p [*Drosophila melanogaster*]
	XP_001069615	PREDICTED: similar to YY1 transcription factor [*Rattus norvegicus*]
	AAY66970	secreted protein [*Ixodes scapularis*]
	XP_785823	PREDICTED: similar to dispatched homolog 1 [*Strongylocentrotus purpuratus*]
Down-regulated genes	XP_678020	hypothetical protein [*Plasmodium berghei*]
	CAE73165	hypothetical protein [*Caenorhabditis briggsae*]

## Data Availability

The data and calculation tools are available from the corresponding author (ritas@ua.pt).

## Supplementary Materials

The following supporting information can be downloaded at: https://www.mdpi.com/article/10.3390/toxics11110918/s1. Figure S1: Details of the experimental design of the multigenerational experiment; Figure S2: Hierarchical clustering of statistically significantly expressed genes of *Daphnia magna*; Table S1: Full list of differentially transcribed genes; Table S2: Full list of differentially transcribed genes following exposure of *Daphnia magna* to carbendazim (CBZ).
