# Supplementary material for: Daphnia magna Multigeneration Exposure to Carbendazim: Gene Transcription Responses
Source: Toxics. 2023 Nov 10;11(11):918. doi: 10.3390/toxics11110918 (PMC10674461; doi:10.3390/toxics11110918)
Supplement: Supplementary file 1 [file toxics-11-00918-s001.zip › Figures S_art5_proof_revised.pdf]

SUPPLEMENTARY MATERIAL

Daphnia magna multigeneration exposure to carbendazim: gene transcription responses

Ana Rita R. Silva[a], Patrícia V. Silva[a], Ana Raquel Soares[b], M. Nazaret González-Alcaraz[c], Cornelis A.M. van Gestel[d], Dick Roelofs[e], Gabriela Moura[b], Amadeu M. V. M. Soares[a] and Susana Loureiro[a]

[a]Department of Biology & CESAM, University of Aveiro, 3810-193, Portugal

[b]Department of Medical Sciences & Institute for Biomedicine (iBiMED), University of Aveiro, 3810-193 Aveiro, Portugal

[c]Department of Agricultural Engineering of the E.T.S.I.A. & Soil Ecology and Biotechnology Unit of the Institute of Plant Biotechnology, Technical University of Cartagena, 30203, Cartagena, Spain

[d]Department of Ecological Science, Faculty of Science, Vrije Universiteit Amsterdam, The Netherlands

[e]Keygene N.V., Agro Business Park 90, 6708 PW Wageningen, The Netherlands

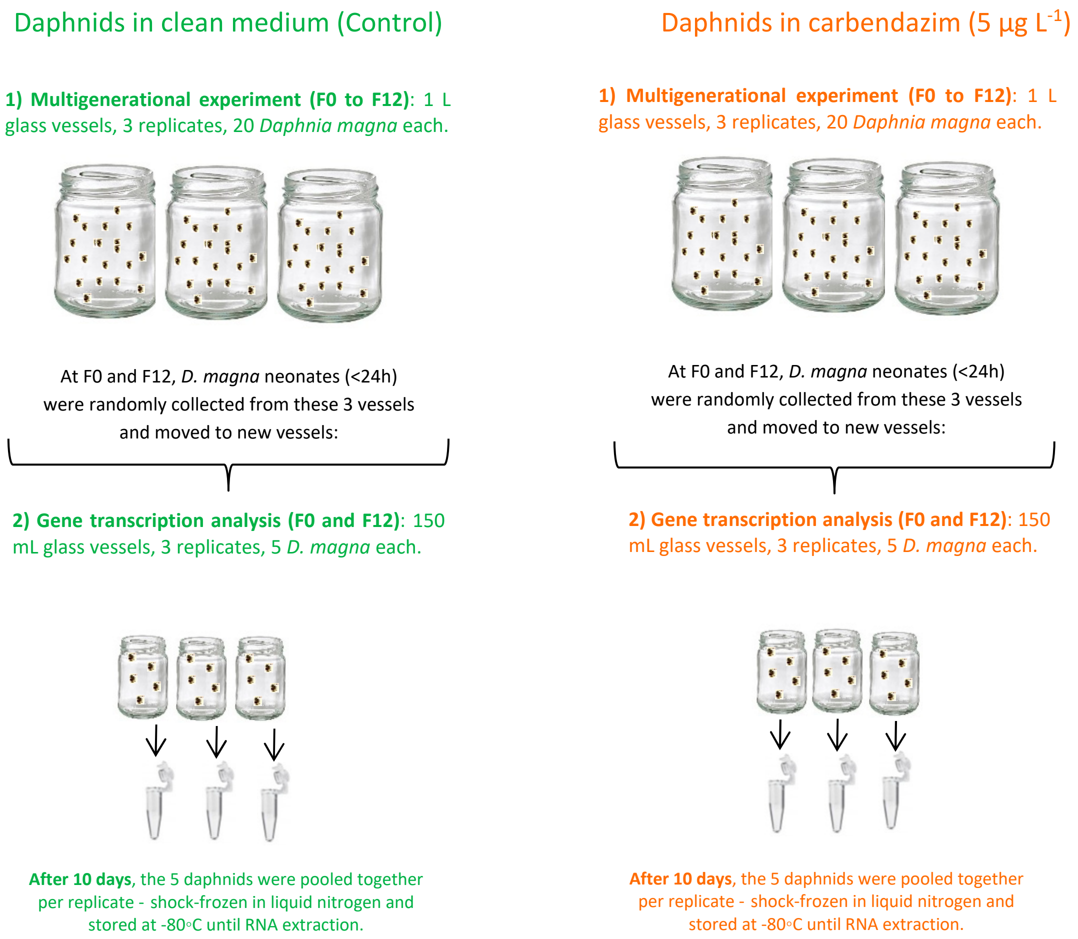

Figure S1. Details of the experimental design of the 1) multigenerational experiment and 2) gene transcription analysis. F represents generation. Note: when moved to new vessels, daphnids were maintained in the same conditions (clean daphnids in clean medium; carbendazim daphnids in carbendazim).

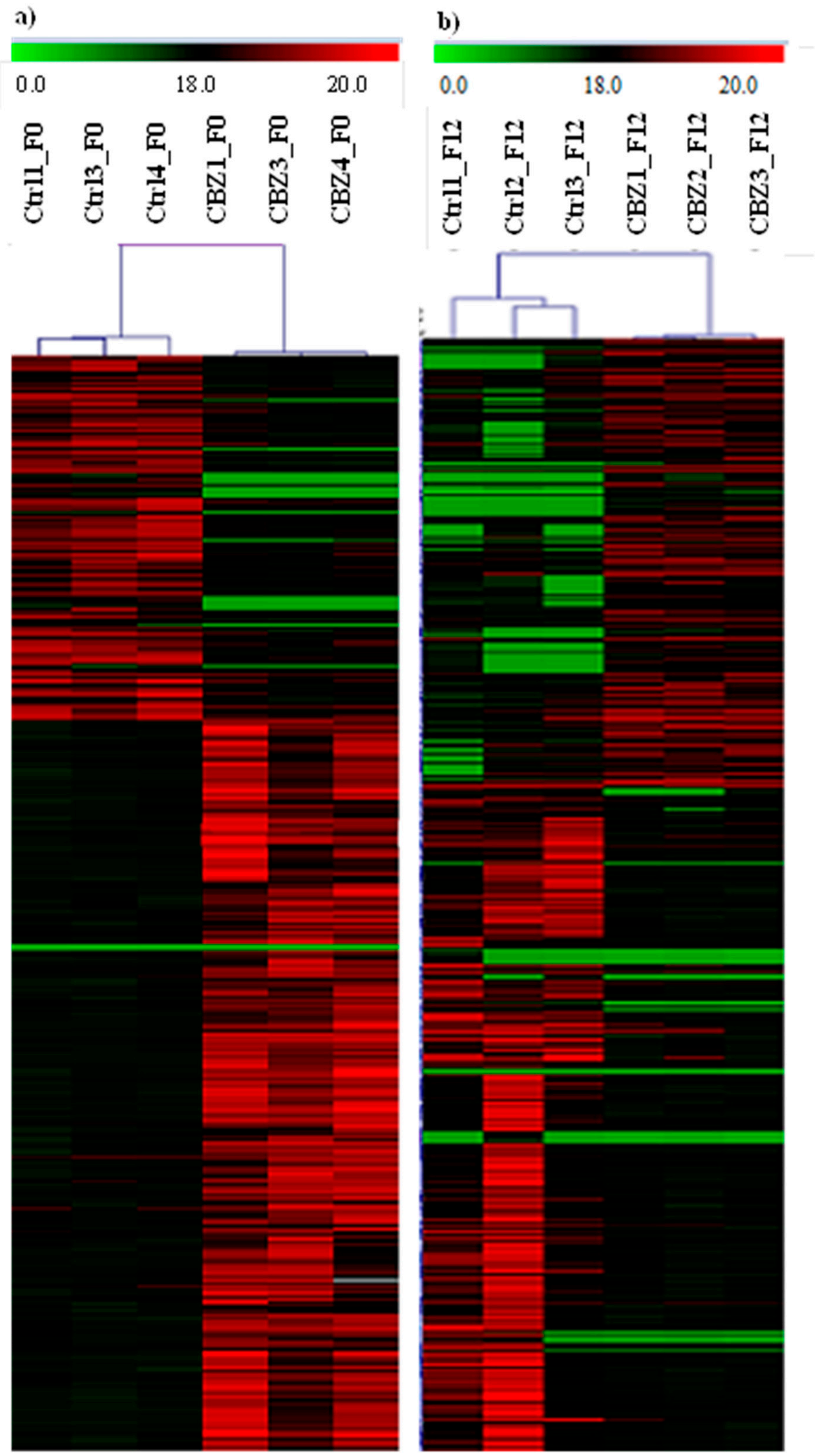

Figure S2. Hierarchical clustering of statistically significantly expressed genes of *Daphnia magna* exposed to 5 µg L^{-1} carbendazim (CBZ) compared with clean medium (controls, Ctrl): **a)** F0 generation, and **b)** F12 generation. A clear separation and clustering between clean medium conditions and carbendazim exposure is observed, with green shading indicating less expressed genes and red shading indicating highly expressed genes.
