# Supplementary material for: Daphnia magna Multigeneration Exposure to Carbendazim: Gene Transcription Responses
Source: Toxics. 2023 Nov 10;11(11):918. doi: 10.3390/toxics11110918 (PMC10674461; doi:10.3390/toxics11110918)
Supplement: Supplementary file 1 [file toxics-11-00918-s001.zip › Table S1_art5.pdf]

Table S1. Full list of differentially transcribed genes. F0 *vs.* F12 generation of *Daphnia magna* are compared: a) clean medium (Clean/Control) (73 up-regulated and 62 down-regulated genes), and b) carbendazim (CBZ) (119 up-regulated and 87 down-regulated genes). Up-regulation was considered whenever the fold change was higher than 1.5, whereas down-regulation was considered whenever the fold change was below -1.5. The arrows refer to up- (↑) or down-regulated (↓) genes.

a) F0 vs. F12 Clean medium

Gene ID	Gene description [species]	Clean (Fold change)
AAV34845	ribosomal protein L34 [*Bombyx mori* (domestic silkworm)]	↑ 16.29
EAA07972	MMSA_ANOGA AGAP002499-PA [*Anopheles gambiae* str. PEST]	↑ 13.23
XP_392882	calcyphosin-like protein-like [*Apis mellifera* (honey bee)]	↑ 12.78
AAF86906	triose phosphate/phosphate translocator precursor [*Mesembryanthemum crystallinum*]	↑ 12.51
AAQ22478	PlexB Plexin B [*Drosophila melanogaster* (fruit fly)]	↑ 11.79
NP_652184	oxidase. Probable cytochrome c oxidase subunit 7A, mitochondrial [*Drosophila melanogaster*]	↑ 11.62
AAV91371	hypothetical protein 3 [*Lonomia obliqua*]	↑11.45
CAE56378	Hypothetical protein CBG24057 [*Caenorhabditis briggsae*]	↑ 10.48
XP_700569	PREDICTED: similar to alpha-2-macroglobulin receptor [*Danio rerio*]	↑ 10.43
XP_393411	ubiquitin-conjugating enzyme E2 variant 2-like [*Apis mellifera* (honey bee)]	↑ 9.78
XP_797717	Golgi SNAP receptor complex member 1 [*Strongylocentrotus purpuratus* (purple sea urchin)]	↑ 9.72
AAV63979	cathepsin L1 precursor [*Artemia parthenogenetica*]	↑ 9.63
XP_605435	PREDICTED: similar to exophilin 5 [*Bos taurus*]	↑ 9.62
XP_392758	S-phase kinase-associated protein 1 [*Apis mellifera* (honey bee)]	↑ 9.54
AAC05908	cytochrome oxidase subunit II [*Pieris rapae*]	↑ 9.14
EAR94332	hypothetical protein TTHERM_00049470 [*Tetrahymena thermophila*]	↑ 9.11
EAT38914	AAEL009235-PA [*Aedes aegypti* (yellow fever mosquito)]	↑ 9.02
XP_968978	PREDICTED: similar to intracellular membrane-associated calcium-independent phospholipase A2 gamma [*Tribolium castaneum*]	↑ 9.01
XP_001072503	PREDICTED: similar to putative MAPK activating protein PM20,PM21 isoform 1 [*Rattus norvegicus*]	↑ 8.75
DAA04586	TPA: TPA_inf: RTN3-A2 [*Oncorhynchus mykiss*]	↑ 8.19
XP_757724	G-protein coupled receptor [*Ustilago maydis*]	↑ 7.92
AAI16802	melanoma antigen family B, 18 [*Mus musculus* (house mouse)]	↑ 7.89
ZP_01120123	hypothetical protein RB2501_07115 [*Robiginitalea biformata*]	↑ 7.81
NP_571006	solute carrier family 39 (zinc transporter), member 7 [*Danio rerio*]	↑ 7.81
XP_623241	growth hormone-inducible transmembrane protein-like [*Apis mellifera* (honey bee)]	↑ 7.78
BAB21109	elongation factor 1 delta [*Bombyx mori* (domestic silkworm)]	↑ 7.72
AAM44044	ferritin 2 [*Apriona germari*]	↑ 7.64
XP_967422	DNA replication licensing factor Mcm7 [*Tribolium castaneum* (red flour beetle)]	↑ 7.32
ABD19264	cytochrome b [*Daphnia pulex*]	↑ 7.32
AAL27467	TRL10 [human herpesvirus 5]	↑ 7.02
XP_534308	immunoglobulin receptor superfamily, protein phosphatase, immunoglobulin superfamily cell adhesion molecule [*Canis familiaris*]	↑ 6.98
CAG08737	unnamed protein product [*Tetraodon nigroviridis*]	↑ 6.66
XP_756287	family/subfamily: TRNA-DIHYDROURIDINE(16/17) SYNTHASE [NAD(P)(+)]-LIKE (PTHR11082:SF5) [*Ustilago maydis*]	↑ 6.64
CAF89999	unnamed protein product [*Tetraodon nigroviridis*]	↑ 6.53
AAC28351	cytochrome P450 [*Homarus americanus*]	↑ 6.40
NP_648180	CG13675-PA [*Drosophila melanogaster*]	↑ 6.31
AAS93718	CG30022 [*Drosophila melanogaster* (fruit fly)]	↑ 6.17
BAD32189	Nup160 nucleoporin 160 [*Mus musculus* (house mouse)]	↑ 6.13
XP_455853	hypothetical protein [*Kluyveromyces lactis*]	↑ 5.93
NP_503838	G-protein coupled receptor. [*Caenorhabditis elegans*]	↑ 5.77
CAJ19121	vitellogenin receptor [*Blattella germanica*]	↑ 5.54
ZP_01117305	Zebrafish DNA sequence from clone CH211-69O18 in linkage group 16, complete sequence	↑ 5.53

XP_394275	nucleolar GTP-binding 1-like protein [*Apis mellifera* (honey bee)]	↑ 5.35
EAA08205	AGAP002490-PA [*Anopheles gambiae*]	↑ 5.28
CAG01996	unnamed protein product [*Tetraodon nigroviridis*]	↑ 5.23

Gene ID	Gene description [species]	Clean (Fold change)
AAH18737	brain protein I3 [*Homo sapiens* (human)]	↑ 5.09
AAH62838	hydroxysteroid dehydrogenase like 2 [*Danio rerio* (zebrafish)]	↑ 5.03
CAG01937	unnamed protein product [*Tetraodon nigroviridis*]	↑ 5.02
XP_973337	homocysteine-responsive endoplasmic reticulum-resident ubiquitin-like domain member 2 protein [*Tribolium castaneum* (red flour beetle)]	↑ 4.85
EAA05974	ribosomal protein S3a [*Tribolium castaneum* (red flour beetle)]	↑ 4.72
CAG05006	unnamed protein product [*Tetraodon nigroviridis*]	↑ 4.70
XP_966633	histone deacetylase Rpd3 *[Tribolium castaneum* (red flour beetle)].	↑ 4.54
CAJ02875	hypothetical protein [*Leishmania major*]	↑ 4.40
AAM09808	Sec31p [*Yarrowia lipolytica*]	↑ 4.10
ZP_01231398	hypothetical protein CdifQ_02001672 [*Clostridium difficile*]	↑ 4.06
XP_360078	predicted protein [*Magnaporthe grisea*]	↑ 3.97
XP_953782	TA16735 hypothetical protein [*Theileria annulata*]	↑ 3.89
XP_827581	hypothetical protein [*Trypanosoma brucei brucei*]	↑ 3.82
ABA25160	transposase [*Anabaena variabilis*]	↑ 3.47
YP_511438	FAD dependent oxidoreductase [*Jannaschia* sp.]	↑ 3.47
EAR85282	phospholipid-translocating P-type ATPase, flippase family protein [*Tetrahymena thermophila*]	↑ 3.35
XP_973543	polypeptide N-acetylgalactosaminyltransferase 5 [*Tribolium castaneum* (red flour beetle)]	↑ 3.21
XP_623167	PREDICTED: similar to ENSANGP00000026584 [*Apis mellifera*]	↑ 3.16
XP_972276	dolichyl pyrophosphate Man9GlcNAc2 alpha-1,3-glucosyltransferase [*Tribolium castaneum* (red flour beetle)]	↑ 3.15
EAT43025	AAEL005513-PA [*Aedes aegypti* (yellow fever mosquito)]	↑ 2.97
CAF23258	hypothetical protein [*Candidatus Protochlamydia amoebophila*]	↑ 2.90
AAX55746	low mass masquerade-like protein [*Pacifastacus leniusculus*]	↑ 2.62
EAA04645	AGAP007365-PA [*Anopheles gambiae*]	↑ 2.61
XP_855127	PREDICTED: hypothetical protein XP_850034 [*Canis familiaris*]	↑ 2.51
YP_547797	hypothetical protein [*Polaromonas* sp.]	↑ 2.26
AAM12270	GH12942p [*Drosophila melanogaster*]	↑ 1.93
EAL28826	Dpse\GA19957 [*Drosophila pseudoobscura pseudoobscura*]	↑ 1.85
XP_393169	beta-catenin-like protein 1-like [*Apis mellifera* (honey bee)]	↑ 1.76

Gene ID	Gene description [species]	Clean (Fold change)
AAH77956	dymeclin [*Xenopus laevis* (African clawed frog)]	↓ -1.97
XP_001071588	PREDICTED: similar to keratin associated protein 10-10 isoform 1 [*Rattus norvegicus*]	↓ -2.04
XP_688421	PREDICTED: similar to peroxisomal biogenesis factor 14 [*Danio rerio*]	↓ -2.24
AAZ75599	CRISP-ENH2 [*Enhydris polylepis*]	↓ -2.41
AAK52091	Cth cystathionine gamma-lyase [*Rattus norvegicus* (Norway rat)]	↓ -2.48
XP_813347	UDP-glucoronosyl and UDP-glucosyl transferase [*Trypanosoma cruzi*]	↓ -2.53
XP_644052	thioredoxin peroxidase [*Dictyostelium discoideum AX4*]	↓ -2.57
EAA04524	AGAP007120-PA [*Anopheles gambiae str. PEST*]	↓ -2.60
NP_200066	family/subfamily: Dentin sialophosphoprotein-related protein [*Arabidopsis thaliana*]	↓ -2.67
XP_640534	GMP synthetase [*Dictyostelium discoideum*]	↓ -2.91
CAE73165	Hypothetical protein CBG20561 [*Caenorhabditis briggsae*]	↓ -3.05
XP_799186	similar to Angiotensin-converting enzyme, testis-specific isoform precursor (ACE-T) (Dipeptidyl carboxypeptidase I) (Kininase II) [*Strongylocentrotus purpuratus* (purple sea urchin)]	↓ -3.16
XP_746198	hypothetical protein [*Plasmodium chabaudi chabaudi*]	↓ -3.17
CAC95124	TIR/NBS/LRR protein [*Populus deltoides*]	↓ -3.37
Q8WPJ2	FSU_1166 pseudo [*Fibrobacter succinogenes*]	↓ -3.41
ABD33303	hypothetical protein MtrDRAFT_AC158502g12v1 [*Medicago truncatula*]	↓ -3.76
AAL05973	peroxinectin [*Penaeus monodon*]	↓ -3.88
EAS35804	CIMG_01158 hypothetical protein [*Coccidioides immitis RS*]	↓ -4.36
XP_971851	PREDICTED: similar to NADH-ubiquinone oxidoreductase 42 kDa subunit,	↓ -4.53

	mitochondrial precursor (Complex I-42KD) (CI-42KD) [*Tribolium castaneum*]	
AAU18227	hypothetical protein [*Bacillus cereus*]	↓ -4.92
ABG63070	50S ribosomal protein L2 [*Chelativorans* sp.]	↓ -4.50
XP_765680	hypothetical protein TP01_0153 [*Theileria parva*]	↓ -6.88
XP_785823	PREDICTED: similar to dispatched homolog 1 [*Strongylocentrotus purpuratus*]	↓ -8.52
BAB95816	truncated hypothetical protein, similar to integrase [*Staphylococcus aureus*]	↓ -11.03

Gene ID	Gene description [species]	Clean (Fold change)
EAT48380	AAEL000585-PA [*Aedes aegypti* (yellow fever mosquito)]	↓ -15.29
XP_392710	vam6/Vps39-like protein-like [*Apis mellifera* (honey bee)]	↓ -21.04
AAT61403	prolyl oligopeptidase [*Bacillus thuringiensis*]	↓ -2187.35
CAG04404	unnamed protein product [*Tetraodon nigroviridis*]	↓ -5409.11
AAZ45366	Daro_0609 peptidase M23B [*Dechloromonas aromatica*]	↓ -9110.36
NP_922637	Os10g0550300 [*Oryza sativa Japonica Group*]	↓ -10936.32
AAH90827	protease, serine, 60.2 [*Danio rerio* (zebrafish)]	↓ -15512.09
XP_465194	putative speckle-type POZ protein [*Oryza sativa* (japonica cultivar-group)]	↓ -16218.71
CAJ28140	CD9 antigen [*Dasyatis akajei*]	↓ -18236.02
NP_014128	non-motor actin binding protein [*Saccharomyces cerevisiae*]	↓ -21296.16
BAE56654	unnamed protein product [*Aspergillus oryzae*]	↓ -24885.83
AAH74846	transmembrane protease, serine 3 [*Homo sapiens* (human)]	↓ -25489.30
AAY54998	IP06749p [*Drosophila melanogaster*]	↓ -27711.51
P36308	Outer capsid protein VP4 (Hemagglutinin) [*Caenorhabditis elegans*]	↓ -28918.30
XP_624252	histone-lysine N-methyltransferase trr [*Apis mellifera* (honey bee)]	↓ -31266.20
CAG76660	ECA_RS18580 hypothetical protein [*Pectobacterium atrosepticum*]	↓ -33727.01
AAS53787	AGOS_AFR416C AFR416Cp [*Ashbya gossypii*]	↓ -37790.57
CAD51508	PFE0715w asparagine-tRNA ligase, putative [*Plasmodium falciparum 3D7*]	↓ -39926.89
ZP_00637578	hypothetical protein SfriDRAFT_3544 [*Shewanella frigidimarina*]	↓ -45161.62
AAM74161	Pax-6 protein [*Euprymna scolopes*]	↓ -46398.67
AAW88416	Serpentine receptor, class bc (class b-like) protein 36 [*Caenorhabditis elegans*]	↓ -53891.71
ABG52939	Tery_3906 hypothetical protein [*Trichodesmium erythraeum IMS101*]	↓ -60067.61
EAL26781	Dpse\GA20714 [*Drosophila pseudoobscura pseudoobscura*]	↓ -66170.96
EAR91277	transmembrane protein, putative [*Tetrahymena thermophila SB210*]	↓ -74204.27
NP_008814	NADH dehydrogenase subunit 6 [*Mustelus manazo* (starspotted smooth-hound)]	↓ -79749.44
XP_736601	hypothetical protein [*Plasmodium chabaudi chabaudi*]	↓ -82206.53
AAZ69264	MBAR_RS01565 hypothetical protein [*Methanosarcina barkeri*]	↓ -85847.78
ABG52453	Tery_3351 peptidase M23B [*Trichodesmium erythraeum*]	↓ -87307.18
XP_665805	hypothetical protein [*Cryptosporidium hominis*]	↓ -88792.28
AAH88813	lectin, galactoside-binding, soluble, 9B [*Xenopus (Silurana) tropicalis* (western clawed frog)]	↓ -114447.56
AAT64428	pMGA 1.4 [*Mycoplasma gallisepticum*]| haemagglutinin homologue	↓ -114453.63
EAT40951	AAEL007376-PA [*Aedes aegypti* (yellow fever mosquito)]	↓ -119236.95
YP_476405	CYB_0141 hypothetical protein [Synechococcus sp. JA-2-3B'a(2-13)]	↓ -129500.50
CAA17132	putative protein [*Arabidopsis thaliana*]	↓ -137110.85
CAD67790	double stranded RNA-activated protein kinase 1 [*Tetraodon nigroviridis*]	↓ -147548.17
ZP_00370063	conserved hypothetical protein [*Campylobacter upsaliensis*]	↓ -151124.91
XP_827078	hypothetical protein [*Trypanosoma brucei brucei*]	↓ -171478.98
BAD94515	peroxisome proliferator-activated receptor gamma [*Oncorhynchus keta*]	↓ -194969.42

b) F0 vs. F12 carbendazim

Gene ID	Gene description [species]	CBZ (fold change)
XP_700169	PREDICTED: similar to conserved hypothetical protein [*Danio rerio*]	↑ 83178.68
ZP_01011697	outer membrane lipoprotein Blc [*Rhodobacterales bacterium*]	↑ 75647.24
XP_500810	SEC16, ISOFORM F; hypothetical protein [*Yarrowia lipolytica*]	↑ 65357.29
AAM51523	Hypothetical protein C28G1.6 [*Caenorhabditis elegans*]	↑ 38447.13
XP_765680	hypothetical protein TP01_0153 [*Theileria parva*]	↑ 16642.07
AAQ75727	NADH dehydrogenase I [*Errhomus variabilis*]	↑ 13302.91
XP_784306	PREDICTED: similar to placental protein 11 related [*Strongylocentrotus purpuratus*]	↑ 6822.25
AAN73266	polyprotein [Tick-borne encephalitis virus]	↑ 5696.41

AAH81106	MGC83377 protein [*Xenopus laevis*]; solute carrier family 5 (sodium/glucose cotransporter), member 2	↑14.93
CAD43195	disrupted in schizophrenia 1 protein [*Danio rerio*]	↑ 10.50
EAL25204	GA13016-PA [*Drosophila pseudoobscura*]	↑ 9.75
XP_829305	tyrosine phosphatase [*Trypanosoma brucei*]	↑ 8.79
AAK41879	Conserved hypothetical protein [*Sulfolobus solfataricus*]	↑ 8.10
ZP_00120785	COG2378: Predicted transcriptional regulator [*Bifidobacterium longum*]	↑ 7.77
XP_874527	PREDICTED: similar to keratin associated protein 10-7 [*Bos taurus*]	↑ 7.52
EAT35209	4-aminobutyrate aminotransferase [*Aedes aegypti*]	↑ 7.41

Gene ID	Gene description [species]	CBZ (fold change)
EAL28674	GA15521-PA [*Drosophila pseudoobscura*]	↑ 7.30
XP_971017	PREDICTED: zinc transporter ZIP11 isoform X2 [*Tribolium castaneum*]	↑ 7.15
XP_973490	PREDICTED: similar to CG2918-PA [*Tribolium castaneum*]; hypoxia up-regulated protein 1	↑ 7.07
NP_039078	Ankyrin repeat gene family protein [*Fowlpox virus*]	↑ 6.68
EAA10981	ENSANGP00000011510 [*Anopheles gambiae*]	↑ 6.51
CAA73128	Irp3 protein [*Yersinia enterocolitica*]	↑ 6.43
XP_974201	39S ribosomal protein L44, mitochondrial	↑ 6.20
AAV34884	ribosomal protein S27 [*Bombyx mori*]	↑ 6.12
XP_500452	hypothetical protein [*Yarrowia lipolytica*]	↑ 6.08
XP_421566	PREDICTED: similar to MAWD binding protein (Unknown protein 32 from 2D-page of liver tissue) [*Gallus gallus*]	↑ 5.74
EAT34816	glycine cleavage system h protein [*Aedes aegypti*]	↑ 5.60
AAC27659	tryptophan oxygenase [*Anopheles gambiae*]	↑ 5.22
XP_975769	cytochrome b-c1 complex subunit 2, mitochondrial [*Tribolium castaneum*]	↑ 5.13
ABF94587	Cystatin, putative [*Oryza sativa* (japonica cultivar-group)]	↑ 4.92
XP_974675	zinc transporter ZIP1 [*Tribolium castaneum*]	↑ 4.82
XP_361380	hypothetical protein MG03854.4 [*Magnaporthe grisea* 70-15]	↑ 4.74
AAT74669	cysteine-rich secreted protein 3 [*Mesocestoides vogae*]	↑ 4.64
XP_969056	ubiquitin carboxyl-terminal hydrolase 14 [*Tribolium castaneum* (red flour beetle)]	↑ 4.73
XP_966449	PREDICTED: similar to CG10092-PA [*Tribolium castaneum*]	↑ 4.72
EAT36570	alcohol dehydrogenase [*Aedes aegypti*]	↑ 4.72
EAR99683	Pre-mRNA cleavage complex II protein Clp1, putative; hypothetical protein TTHERM_00590200 [*Tetrahymena thermophila* SB210]	↑ 4.71
XP_001102209	PREDICTED: similar to Methylmalonyl-CoA epimerase, mitochondrial precursor (DL-methylmalonyl-CoA racemase) isoform 1 [*Macaca mulatta*]	↑ 4.68
AAZ14281	proteophosphoglycan 5 [*Leishmania* major strain Friedlin]	↑ 4.67
XP_809835	subtilisin-like serine peptidase [*Trypanosoma cruzi* strain CL Brener]	↑ 4.62
ABA02335	NADH dehydrogenase subunit 2 [*Daphnia magna*]	↑ 4.59
XP_797537	PREDICTED: similar to membrane protein mKirre [*Strongylocentrotus purpuratus*]	↑ 4.57
XP_967940	PREDICTED: similar to sideroflexin 2 [*Tribolium castaneum*]	↑ 4.57
ABF51294	NADPH-specific isocitrate dehydrogenase	↑ 4.51
YP_473323	ORF135 peptide [Hyphantria cunea nucleopolyhedrovirus]	↑ 4.51
EAS03884	Viral A-type inclusion protein repeat containing protein [*Tetrahymena thermophila*]	↑ 4.43
CAG31427	annexin A11	↑ 4.39
AAH90678	hypothetical protein [*Danio rerio*]	↑ 4.36
XP_646129	putative Nek family protein kinase [*Dictyostelium discoideum*]	↑ 4.31
XP_393344	PREDICTED: similar to hypothetical protein 18 [*Apis mellifera*]	↑ 4.27
CAC44629	deafness dystonia protein [*Takifugu rubripes*]	↑ 4.18
AAH95821	Snx9l protein; monooxygenase, DBH-like 1, like [*Danio rerio* (zebrafish)]	↑ 4.17
EAS36540	DNA polymerase epsilon catalytic subunit A [*Coccidioides immitis*]	↑ 4.10
AAR37644	molybdopterin-guanine dinucleotide biosynthesis protein [uncultured bacterium 439]	↑ 4.07
AAR04057	sulfatase FP1b [*Danio rerio*]	↑ 4.05
AAX24666	SJCHGC03634 protein [*Schistosoma japonicum*]	↑ 4.04
XP_392990	PREDICTED: similar to CG8385-PB, isoform B [*Apis mellifera*]	↑ 4.04
XP_397355	transmembrane protein 64-like [*Apis mellifera*]	↑ 3.99
AAS94231	legumain-like protease precursor [*Ixodes ricinus*]	↑ 3.97
BAC98829	F-box WD40 protein [*Labidochromis caeruleus*]	↑ 3.89
XP_791484	pREDICTED: similar to beta-1,4-mannosyltransferase, partial [*Strongylocentrotus purpuratus*]	↑ 3.84
XP_966285	DNA polymerase epsilon catalytic subunit A [*Plasmodium falciparum*]	↑ 3.83

BAB74771	alr3072 [*Nostoc* sp. PCC 7120]	↑ 3.81
XP_967132	PREDICTED: similar to CG6016-PB, isoform B isoform 1 [*Tribolium castaneum*]	↑ 3.81
XP_502986	hypothetical protein [*Yarrowia lipolytica*]	↑ 3.79
EAT45353	conserved hypothetical protein [*Aedes aegypti*]	↑ 3.78
ABA94886	Leucine Rich Repeat family protein, expressed [*Oryza sativa* (japonica cultivar-group)]	↑ 3.76
XP_001075301	PREDICTED: similar to CG13731-PA [Rattus norvegicus]	↑ 3.75
AAH77956	dymeclin [*Xenopus laevis* (African clawed frog)]	↑ 3.71
XP_623472	PREDICTED: similar to ENSANGP00000024947 [*Apis mellifera*]	↑ 3.68
XP_975592	programmed cell death protein 6 [*Tribolium castaneum* (red flour beetle)]; PREDICTED: similar to CG40410-PA.3 [*Tribolium castaneum*]	↑ 3.67
XP_829735	hypothetical protein Tb11.01.8530 [*Trypanosoma brucei* TREU927]	↑ 3.63
Gene ID	Gene description [species]	CBZ (fold change)
XP_541754	guanyl-nucleotide exchange factor [*Canis familiaris*]	↑ 3.58
XP_393519	PREDICTED: similar to fatty acid-biding protein [*Apis mellifera*]	↑ 3.56
XP_971885	PREDICTED: similar to CG9762-PA [*Tribolium castaneum*]	↑ 3.56
EAL34060	GA18149-PA [*Drosophila pseudoobscura*]	↑ 3.50
ZP_01103786	conserved hypothetical protein [gamma proteobacterium KT 71]	↑ 3.47
XP_541851	PREDICTED: similar to Stabilin-1 precursor (FEEL-1 protein) (MS-1 antigen) [*Canis familiaris*]	↑ 3.45
XP_393525	PREDICTED: similar to GA20008-PA [*Apis mellifera*]	↑ 3.44
AAH24612	basic transcription factor 3-like 4 [*Mus musculus* (house mouse)]	↑ 3.43
EAT40746	conserved hypothetical protein [*Aedes aegypti*]	↑ 3.37
XP_623417	PREDICTED: similar to ENSANGP00000021084 [*Apis mellifera*]	↑ 3.35
AAK25797	delta-9 desaturase 3 [*Acheta domesticus*]	↑ 3.31
CAJ17256	ribosomal protein L10Ae [*Biphyllus lunatus*]	↑ 3.25
AAH42230	Ribosomal protein S1a protein [*Xenopus laevis*]	↑ 3.23
ZP_01376161	hypothetical protein Ccur5_01001129 [*Campylobacter curvus* 525.92]	↑ 3.16
XP_001076360	PREDICTED: similar to retinoblastoma binding protein 6 isoform 1 isoform 2 [*Rattus norvegicus*]	↑ 3.16
XP_971417	ATP synthase mitochondrial F1 complex assembly factor 1 [*Tribolium castaneum* (red flour beetle)]	↑ 3.10
CAG00408	unnamed protein product [*Tetraodon nigroviridis*]	↑ 3.05
ZP_01029146	hypothetical protein Badol_01000893 [*Bifidobacterium adolescentis*]	↑ 2.99
XP_691505	PREDICTED: similar to obscurin, cytoskeletal calmodulin and titin-interacting RhoGEF [*Danio rerio*]	↑ 2.98
CAE68115	Hypothetical protein CBG13758 [*Caenorhabditis briggsae*]	↑ 2.85
EAA05425	ENSANGP00000012700 [*Anopheles gambiae* str. PEST]	↑ 2.81
EAT44525	Niemann-Pick Type C-2, putative [*Aedes aegypti*]	↑ 2.79
AAS55566	MST84DC [*Drosophila simulans*]	↑ 2.78
NP_079004	succinyl-CoA:glutarate-CoA transferase [*Homo sapiens* (human)]	↑ 2.66
CAI86921	conserved protein of unknown function ; putative membrane protein [*Pseudoalteromonas haloplanktis* TAC125]	↑ 2.65
AAH44037	Cg8286-prov protein [*Xenopus laevis*]	↑ 2.65
YP_258141	hypothetical protein PFL_1010 [*Pseudomonas fluorescens* Pf-5]	↑ 2.62
XP_549134	PREDICTED: similar to sushi-repeat-containing protein, X-linked 2 [*Canis familiaris*]	↑ 2.58
AAQ23388	Rab7 [*Aiptasia pulchella*]	↑ 2.56
EAA12881	AGAP007952-PA [*Anopheles gambiae str. PEST*]	↑ 2.55
BAB05837	BH2118 [*Bacillus halodurans* C-125]	↑ 2.53
XP_636901	RING zinc finger-containing protein [*Dictyostelium discoideum AX4*]	↑ 2.53
ZP_00131989	COG3106: Predicted ATPase [*Haemophilus somnus* 2336]	↑ 2.44
EAT48619	serine/threonine-protein kinase vrk [*Aedes aegypti*]	↑ 2.40
CAC95124	TIR/NBS/LRR protein [*Populus deltoides*]	↑ 2.38
XP_699475	REDICTED: similar to Sympk protein [*Danio rerio*]	↑ 2.31
XP_980893	PREDICTED: hypothetical protein LOC442837 [*Mus musculus*]	↑ 2.27
XP_511735	PREDICTED: similar to hypothetical protein FLJ22175 [*Pan troglodytes*]	↑ 2.19
EAA00702	AGAP011988-PA *[Anopheles gambiae* str. PEST]	↑ 2.15
XP_625439	insulinase like peptidase [*Cryptosporidium parvum* Iowa II]	↑ 2.12
AAD50987	prothymosin a14 [*Homo sapiens*]	↑ 2.10
XP_973265	ribosome biogenesis protein BOP1 homolog [*Tribolium castaneum* (red flour beetle)]	↑ 2.08
EAA13751	AGAP010769-PA	↑ 1.94

NP_649657	Zinc-finger protein [*Drosophila melanogaster* (fruit fly)]	↑ 1.75
EAS00551	hypothetical protein TTHERM_00409040 [*Tetrahymena thermophila* SB210]	↑ 1.73
XP_710938	Potential fungal zinc cluster transcription factor [*Candida albicans*]	↑ 1.73
XP_686827	PREDICTED: similar to Bmp1 protein [*Danio rerio*]	↑ 1.56

Gene ID	Gene description [species]	CBZ (Fold change)
NP_872312	melanoma antigen family B10 [*Homo sapiens* (human)]	↓ -1.57
XP_971465	PREDICTED: similar to Tubulin tyrosine ligase-like protein 2 (Testis-specific protein NYD-TSPG) [*Tribolium castaneum*]	↓ -1.63
AAN36896	conserved Plasmodium protein, unknown function [*Plasmodium falciparum 3D7*]	↓ -1.71
BAE73006	hypothetical protein [*Macaca fascicularis*]	↓ -1.71
EAT47168	serine protease [*Aedes aegypti*]	↓ -1.89

Gene ID	Gene description [species]	CBZ (Fold change)
NP_001026467	serine/arginine-rich splicing factor 11 [*Gallus gallus* (chicken)]	↓ -1.89
NP_523475	Salivary gland secretion 1 CG3047-PA [*Drosophila melanogaster*]	↓ -1.96
XP_678020	Triops cancriformis mitochondrial DNA, complete genome [*Plasmodium berghei*]	↓ -2.04
EAR83186	WD domain, G-beta repeat protein [*Tetrahymena thermophila SB210*]	↓ -2.20
CAE61304	Hypothetical protein CBG05137 [*Caenorhabditis briggsae*]	↓ -2.23
XP_991669	PREDICTED: similar to pericardin CG5700-PB [*Mus musculus*]	↓ -2.70
AAO12215	trypsin [*Aplysina fistularis*]	↓ -3.13
ZP_01223527	4-carboxy-2-hydroxymuconate-6-semialdehyde dehydrogenase [marine gamma proteobacterium HTCC2207]	↓ -3.16
EAS02587	AMP-binding enzyme family protein [*Tetrahymena thermophila* SB210]	↓ -3.43
BAD63461	phage-related protein [*Bacillus clausii* KSM-K16]	↓ -3.48
CAA20431	GTPase regulator (predicted) [*Schizosaccharomyces pombe 972h-*]	↓ -3.70
ZP_01034049	His/Glu/Gln/Arg/opine family ABC transporter, permease protein [*Roseovarius* sp. 217]	↓ -3.71
ZP_01133737	lipase, putative [*Pseudoalteromonas tunicata* D2]	↓ -3.80
AAH85561	Hypothetical protein [*Danio rerio*]	↓ -3.84
XP_969486	PREDICTED: similar to Jagged-1 precursor (Jagged1) [*Tribolium castaneum*]	↓ -4.03
NP_611397	CG15111-PA, isoform A [*Drosophila melanogaster*]	↓ -4.09
XP_971073	AP-1 complex subunit sigma-2 [*Tribolium castaneum* (red flour beetle)]	↓ -4.12
XP_970811	vesicle transport protein SEC20 [Tribolium castaneum (red flour beetle)]	↓ -4.17
XP_624856	BTB/POZ domain-containing protein KCTD5-like [*Apis mellifera* (honey bee)]	↓ -4.18
XP_380554	Hypothetical protein FG00378.1 [*Gibberella zeae* PH-1]	↓ -4.21
XP_392015	PREDICTED: similar to CG31116-PC, isoform C [*Apis mellifera*]	↓ -4.22
XP_426719	PREDICTED: similar to seven in absentia homolog 2; seven in absentia [*Drosophila*] homolog 2 [*Gallus gallus*]	↓ -4.46
ZP_01304133	xylosidase/arabinosidase [*Sphingomonas* sp. SKA58]	↓ -4.54
XP_523482	PREDICTED: hypothetical protein [*Pan troglodytes*]	↓ -4.56
AAD55141	di-domain hemoglobin precursor [*Daphnia pulex*]	↓ -4.61
XP_623750	myotrophin-like [*Apis mellifera* (honey bee)]	↓ -4.85
XP_397115	PREDICTED: similar to ENSANGP00000014264 [*Apis mellifera*]	↓ -4.98
EAL17999	hypothetical protein [*Cryptococcus neoformans var. neoformans B-3501A*]	↓ -4.98
BAE38837	Zranb2 zinc finger, RAN-binding domain containing 2 [*Mus musculus* (house mouse)]	↓ -5.08
XP_995118	PREDICTED: hypothetical protein [*Mus musculus*]	↓ -5.14
AAO27090	CTP synthase [*Buchnera aphidicola* str. Bp (Baizongia pistaciae)]	↓ -5.20
EAA09458	AGAP005124-PA [*Anopheles gambiae str. PEST*]	↓ -5.25
XP_394551	NEDD8-conjugating enzyme UBE2F-like [*Apis mellifera* (honey bee)]	↓ -5.26
XP_549046	PREDICTED: similar to hephaestin isoform a [*Canis familiaris*]	↓ -5.26
EAT46404	conserved hypothetical protein [*Aedes aegypti*]	↓ -5.36
XP_966534	ethanolamine-phosphate cytidylyltransferase [*Tribolium castaneum* (red flour beetle)]	↓ -5.37
EAT43058	AAEL005474-PA [*Aedes aegypti* (yellow fever mosquito)]	↓ -5.40
NP_650751	CG7718 [*Drosophila melanogaster*]	↓ -5.42
AAS48991	UL112/113 [*Human herpesvirus* 5]	↓ -5.47
BAB21109	Ef-1d elongation factor 1 delta [*Bombyx mori* (domestic silkworm)]	↓ -5.51
EAL26005	Dpse\GA14100 [*Drosophila pseudoobscura pseudoobscura*]	↓-5.60
EAT38914	AAEL009235-PA [*Aedes aegypti* (yellow fever mosquito)]	↓ -5.64

CAA84321	Hypothetical protein F45H7.1 [*Caenorhabditis elegans*]	↓ -5.66
XP_455853	unnamed protein product [*Kluyveromyces lactis*]	↓ -5.88
ABB06938	FAD dependent oxidoreductase [*Burkholderia* sp. 383]	↓ -6.10
CAH90002	EIF4A2 eukaryotic translation initiation factor 4A2 [*Pongo abelii* (Sumatran orangutan)]	↓ -6.21
CAK11279	novel protein similar to vertebrate topoisomerase (DNA) II beta 180kDa (TOP2B) [*Danio rerio*]	↓ -6.54
XP_682935	Uncharacterized protein. Subfamily: SOLUTE CARRIER FAMILY 35 MEMBER F1 (PTHR14233:SF10) [*Danio rerio*]	↓ -6.55
XP_796182	PREDICTED: similar to SHQ1 homolog [*Strongylocentrotus purpuratus*]	↓ -6.22
ABF51368	H^+ transporting ATP synthase O subunit [*Bombyx mori* (domestic silkworm)]	↓ -6.64
XP_785816	PREDICTED: similar to muscle Y-box protein YB2 [*Strongylocentrotus purpuratus*]	↓ -6.66
EAS03921	HMG box protein [*Tetrahymena thermophila SB210*]	↓ -6.68
XP_967013	PREDICTED: similar to CG9160-PA, isoform A [*Tribolium castaneum*]	↓ -6.77
ZP_01065094	hypothetical protein MED222_15549 [*Vibrio* sp. MED222]	↓ -6.84
XP_001072503	PREDICTED: similar to putative MAPK activating protein PM20,PM21 isoform 1 [*Rattus norvegicus*]	↓ -6.92

Gene ID	Gene description [species]	CBZ (Fold change)
CAG09120	unnamed protein product [*Tetraodon nigroviridis*]	↓ -7.05
YP_258814	cytochrome c-type biogenesis protein CycH [*Pseudomonas fluorescens* Pf-5]	↓ -7.13
XP_973533	malate dehydrogenase, mitochondrial [*Tribolium castaneum* (red flour beetle)]	↓ -7.14
AAH76191	thoc7 THO complex 7 [*Danio rerio* (zebrafish)]	↓ -7.15
CAF94261	unnamed protein product [*Tetraodon nigroviridis*]	↓ -7.20
CAG05207	unnamed protein product [*Tetraodon nigroviridis*]	↓ -7.30
XP_976221	PREDICTED: similar to CG4944-PB, isoform B isoform 2 [Tribolium castaneum]	↓ -7.33
XP_968064	40S ribosomal protein S3a [*Tribolium castaneum* (red flour beetle)]	↓ -7.36
NP_077791	lipoic acid synthetase [Mus musculus]	↓ -7.49
AAV31414	26S protease regulatory subunit-like protein [*Toxoptera citricida*]	↓ -7.65
AAX28551	SJCHGC05463 protein [*Schistosoma japonicum*]	↓ -7.66
EAA08286	ENSANGP00000017110 [*Anopheles gambiae* str. PEST]	↓ -7.73
CAB60138	putative polyprotein [Wheat yellow mosaic virus]	↓ -8.05
AAN37244	conserved Plasmodium protein, unknown function [*Plasmodium falciparum 3D7*]	↓ -8.09
CAF92521	unnamed protein product [*Tetraodon nigroviridis*]	↓ -8.50
XP_974308	metaxin-2-like [*Tribolium castaneum* (red flour beetle)]	↓ -8.57
XP_624608	renin receptor-like [*Apis mellifera* (honey bee)]	↓ -8.83
YP_052887	ribosomal protein S7 [*Saprolegnia ferax*]	↓ -8.85
AAY60144	oocyte maturation factor Mos [*Anas poecilorhyncha*]	↓ -8.87
XP_623241	growth hormone-inducible transmembrane protein-like [*Apis mellifera* (honey bee)]	↓ -9.53
AAK27862	Hypothetical protein Y37E3.4 [*Caenorhabditis elegans*]	↓ -9.96
ZP_01120123	hypothetical protein RB2501_07115 [Robiginitalea biformata HTCC2501]	↓ -12.89
XP_973543	polypeptide N-acetylgalactosaminyltransferase 5	↓ -14.60
XP_855304	PREDICTED: similar to cyclin K [*Canis familiaris*]	↓ -6377.84
YP_581584	histidine kinase [*Psychrobacter cryohalolentis* K5]	↓ -17088.07
EAM56123	conserved hypothetical protein [*Solibacter usitatus* Ellin]	↓ -49456.71
XP_729076	hypothetical protein PY01338 [*Plasmodium yoelii* yoelii]	↓ -58892.73
