# Supplementary material for: Daphnia magna Multigeneration Exposure to Carbendazim: Gene Transcription Responses
Source: Toxics. 2023 Nov 10;11(11):918. doi: 10.3390/toxics11110918 (PMC10674461; doi:10.3390/toxics11110918)
Supplement: Supplementary file 1 [file toxics-11-00918-s001.zip › Table S2_art5.pdf]

Table S2. Full list of differentially transcribed genes following exposure of *Daphnia magna* to carbendazim (CBZ): a) F0 generation (191 up-regulated and 98 down-regulated genes), and b) F12 generation (53 up-regulated and 66 down-regulated genes). Up-regulation was considered whenever the fold change was higher than 1.5, whereas down-regulation was considered whenever the fold change was below -1.5. The arrows refer to up- (↑) or down-regulated (↓) genes compared to clean medium (control).

a) F0 generation

Gene ID	Gene description [species]	CBZ (Fold change)
T32007	hypothetical protein F36H9.6 [*Caenorhabditis elegans*]	↑ 18.95
XP_973543	polypeptide N-acetylgalactosaminyltransferase 5 [*Tribolium castaneum*]	↑ 18.77
YP_548045	hypothetical protein Bpro_1196, membrane protein [*Polaromonas* sp.]	↑ 18.04
XP_384927	hypothetical protein [*Fusarium graminearum*]	↑ 15.59
XP_966633	histone deacetylase Rpd3 [*Tribolium castaneum* (red flour beetle)]	↑ 14.83
XP_785816	PREDICTED: similar to muscle Y-box protein YB2 [*Strongylocentrotus purpuratus*]	↑ 14.83
YP_052887	rps7 ribosomal protein S7 [*Saprolegnia ferax*]	↑ 14.18
NP_001018342	tas2r203 taste receptor, type 2, member 203 [*Danio rerio* (zebrafish)]	↑ 14.01
XP_393137	UPF0183 protein CG7083-like [*Apis mellifera* (honey bee)]	↑ 13.92
ZP_00592874	GCN5-related N-acetyltransferase [*Prosthecochloris aestuarii*]	↑ 13.72
AAH76191	thoc7 THO complex 7 [*Danio rerio* (zebrafish)]	↑ 13.27
XP_755614	AFUA_2G12830 UDP-glucosyl transferase family protein [*Aspergillus fumigatus*]	↑ 13.07
YP_660772	hypothetical protein [*Pseudoalteromonas atlantica*]	↑ 12.85
XP_392758	S-phase kinase-associated protein 1 [*Apis mellifera* (honey bee)]	↑ 12.75
EAT38914	AAEL009235-PA [*Aedes aegypti* (yellow fever mosquito)]	↑ 12.49
AAV34845	RpL34 ribosomal protein L34 [*Bombyx mori* (domestic silkworm)]	↑ 12.20
CAG10013	unnamed protein product [*Tetraodon nigroviridis*]	↑ 12.18
CAG01937	unnamed protein product [*Tetraodon nigroviridis*]	↑ 12.18
XP_397060	uncharacterized [*Apis mellifera* (honey bee)]	↑ 12.04
AAH62838	hsdl2 hydroxysteroid dehydrogenase like 2 [*Danio rerio* (zebrafish)]	↑ 12.00
BAE38837	Zranb2 zinc finger, RAN-binding domain containing 2 [*Mus musculus* (house mouse)]	↑ 11.90
AAX28551	SJCHGC05463 protein [*Schistosoma japonicum*]	↑ 11.84
XP_623241	growth hormone-inducible transmembrane protein-like [*Apis mellifera* (honey bee)]	↑ 11.75
BAB21109	Ef-1d elongation factor 1 delta [*Bombyx mori* (domestic silkworm)]	↑ 11.59
AAC28351	cytochrome P450 [*Homarus americanus*]	↑ 11.59
XP_455853	hypothetical protein [*Kluyveromyces lactis*]	↑ 11.44
XP_970811	vesicle transport protein SEC20 [*Tribolium castaneum* (red flour beetle)]	↑ 11.42
ZP_01120123	hypothetical protein RB2501_07115 [*Robiginitalea biformata*]	↑ 11.15
AAH41737	eif5 eukaryotic translation initiation factor 5 [*Xenopus laevis* (African clawed frog)]	↑ 10.81
XP_363794	hypothetical protein MG01720.4 [*Magnaporthe grisea*]	↑ 10.58
XP_971073	AP-1 complex subunit sigma-2 [*Tribolium castaneum* (red flour beetle)]	↑ 10.55
AAQ22478	PlexB Plexin B [*Drosophila melanogaster* (fruit fly)]	↑ 10.53
XP_623750	myotrophin-like [*Apis mellifera* (honey bee)]	↑ 10.48
EAA07972	MMSA_ANOGA AGAP002499-PA [*Anopheles gambiae str. PEST*]	↑ 10.37
NP_503838	G-protein coupled receptor. Protein C50H11.13. species: *Caenorhabditis elegans*	↑ 10.36
AAN37244	PF14_0631 conserved Plasmodium protein, unknown function [*Plasmodium falciparum*]	↑ 10.33
ABF51517	legumaturain [*Bombyx mori* (domestic silkworm)]	↑ 10.31
XP_397115	PREDICTED: similar to ENSANGP00000014264 [*Apis mellifera*]	↑ 10.29
ZP_01117305	Zebrafish DNA sequence from clone CH211-69O18 in linkage group 16, complete sequence	↑ 10.21
EAL26005	Dpse\GA14100 [*Drosophila pseudoobscura pseudoobscura*]	↑ 10.13
EAS03921	TTHERM_00455600 HMG box protein [*Tetrahymena thermophila*]	↑ 10.12
EAT48786	AaeL_AAEL000159 AAEL000159-PA [*Aedes aegypti* (yellow fever mosquito)]	↑ 10.04
AAI16802	Mageb18 melanoma antigen family B, 18 [*Mus musculus* (house mouse)]	↑ 9.90
EAA10370	ENSANGP00000025920 [*Anopheles gambiae* str. PEST]	↑ 9.89
XP_624856	BTB/POZ domain-containing protein KCTD5-like [*Apis mellifera* (honey bee)]	↑ 9.87
CAH90002	EIF4A2 eukaryotic translation initiation factor 4A2 [*Pongo abelii* (Sumatran orangutan)]	↑ 9.85
ZP_01233257	hypothetical protein VAS14_10384 [Vibrio angustum]	↑ 9.80
EAA05974	40S ribosomal protein S3a [*Tribolium castaneum* (red flour beetle)]	↑ 9.74

AAY63979	glycerol-3-phosphate dehydrogenase [*Lysiphlebus testaceipes*]	↑ 9.74
XP_479530	putative potassium transporter [*Oryza sativa* (japonica cultivar-group)]	↑ 9.56
Gene ID	Gene description [species]	CBZ (Fold change)
AAS91007	slowmo [*Bombyx mori* (domestic silkworm)]	↑ 9.41
XP_793079	transcription factor AP-1-like [*Strongylocentrotus purpuratus*(purple sea urchin)]	↑ 9.40
XP_968064	40S ribosomal protein S3a [*Tribolium castaneum* (red flour beetle)]	↑ 9.34
NP_652184	oxidase. Probable cytochrome c oxidase subunit 7A, mitochondrial [*Drosophila melanogaster*]	↑ 9.31
XP_850642	PREDICTED: similar to GDP-mannose pyrophosphorylase B isoform 2 isoform 2 [*Canis familiaris*]	↑ 9.28
P25169	Sodium/potassium-transporting ATPase subunit beta (Sodium/potassium-dependent ATPase beta subunit) [*Artemia* sp.] Na+/K+-exchanging ATPase (EC 3.6.3.9) beta chain - brine shrimp	↑ 9.28
XP_397220	Surf1 surfeit 1 [*Apis mellifera* (honey bee)]	↑ 9.27
XP_700569	PREDICTED: similar to alpha-2-macroglobulin receptor [*Danio rerio* (zebrafish)]	↑ 9.27
CAD70781	hypothetical protein [*Neurospora crassa*]	↑ 9.11
XP_953782	TA16735 hypothetical protein [*Theileria annulata strain Ankara*]	↑ 9.06
EAT43245	stretch regulated skeletal muscle protein, putative [*Aedes aegypti*]	↑ 9.02
XP_797717	Golgi SNAP receptor complex member 1 [*Strongylocentrotus purpuratus* (purple sea urchin)]	↑ 8.93
XP_604956	NPAT nuclear protein, ataxia-telangiectasia locus [*Bos taurus* (cattle)]	↑ 8.89
XP_682935	solute carrier family 35 member F1 [*Danio rerio*]	↑ 8.86
ZP_01065094	hypothetical protein MED222_15549 [*Vibrio* sp.]	↑ 8.79
XP_751302	AFUA_6G14280 flavin-binding monooxygenase-like protein [*Aspergillus fumigatus*]	↑ 8.75
EAA08286	ENSANGP00000017110 [*Anopheles gambiae* str]	↑ 8.62
BAD18123	DESAT4 fatty acid desaturase [*Bombyx mori* (domestic silkworm)]	↑ 8.57
EAR91724	TTHERM_00396960 kinase domain protein [*Tetrahymena thermophila*]	↑ 8.54
CAF94261	unnamed protein product [*Tetraodon nigroviridis*]	↑ 8.35
AAC79426	phosphate transport protein [*Choristoneura fumiferana*]	↑ 8.30
ZP_01223527	4-carboxy-2-hydroxymuconate-6-semialdehyde dehydrogenase [marine gamma proteobacterium]	↑ 8.28
CAE67987	Hypothetical protein CBG13597 [*Caenorhabditis briggsae*]	↑ 8.15
AAN79130	Bacteriophage N4 adsorption protein B [*Escherichia coli*]	↑ 8.06
EAA07158	AgaP_AGAP010476 AGAP010476-PA [*Anopheles gambiae str. PEST*]	↑ 8.05
XP_624608	renin receptor-like [*Apis mellifera* (honey bee)]	↑ 8.00
AAK27862	Hypothetical protein Y37E3.4 [*Caenorhabditis elegans*]	↑ 8.00
XP_791551	cytochrome c1, heme protein, mitochondrial [*Strongylocentrotus purpuratus* (purple sea urchin)]	↑ 7.97
XP_973533	malate dehydrogenase, mitochondrial [*Tribolium castaneum* (red flour beetle)]	↑ 7.80
ZP_00510523	Cell division FtsK/SpoIIIE protein [*Clostridium thermocellum*]	↑ 7.60
BAD63461	phage-related protein [*Bacillus clausii*]	↑ 7.59
XP_967013	PREDICTED: similar to CG9160-PA, isoform A [*Tribolium castaneum*]	↑ 7.59
XP_966534	ethanolamine-phosphate cytidylyltransferase [*Tribolium castaneum* (red flour beetle)]	↑ 7.58
CAF97221	unnamed protein product [*Tetraodon nigroviridis*]	↑ 7.55
AAX57282	CT099 [*Lycopersicon peruvianum*]	↑ 7.53
XP_974308	metaxin-2-like [*Tribolium castaneum* (red flour beetle)]	↑ 7.48
NP_077791	Lipoyl synthase, mitochondrial [*Mus musculus*]	↑ 7.47
XP_995118	PREDICTED: similar to CG13957-PA [*Mus musculus*]	↑ 7.45
XP_392882	calcyphosin-like protein-like [*Apis mellifera* (honey bee)]	↑ 7.38
XP_967422	DNA replication licensing factor Mcm7 [*Tribolium castaneum* (red flour beetle)]	↑ 7.34
AAY60144	oocyte maturation factor Mos [*Anas poecilorhyncha*]	↑ 7.30
ZP_00134041	COG1444: Predicted P-loop ATPase fused to an acetyltransferase [*Actinobacillus pleuropneumoniae*]	↑ 7.26
XP_679830	hypothetical protein [*Plasmodium berghei ANKA*]	↑ 7.25
NP_723776	Vha68-2 CG3762-PC, isoform C; ATP synthase, anion channel, ligand-gated ion channel, DNA binding protein, hydrolase. Subfamily: V-type proton atpase catalytic subunit A (PTHR15184:SF7). [*Drosophila melanogaster*]	↑ 7.19
EAS00570	cyclic nucleotide-binding domain protein [*Tetrahymena thermophila*]	↑ 7.09
XP_624674	translocon-associated protein subunit gamma-like [*Apis mellifera* (honey bee)]	↑ 7.04
XP_393411	ubiquitin-conjugating enzyme E2 variant 2-like [*Apis mellifera* (honey bee)]	↑ 6.98
XP_623978	PREDICTED: similar to ENSANGP00000011134 [*Apis mellifera* (honey bee)]	↑ 6.93
AAI14116	Solute carrier family 25 (mitochondrial thiamine pyrophosphate carrier), member 19 [*Bos taurus* (cattle)]	↑ 6.87
AAO27090	CTP synthase [*Buchnera aphidicola* str]	↑ 6.82
XP_001072503	PREDICTED: similar to putative MAPK activating protein PM20,PM21 isoform 1 [*Rattus norvegicus*]	↑ 6.79

EAA12371	AGAP008234-PA [*Anopheles gambiae str. PEST*]	↑ 6.60
ABF51368	H^+ transporting ATP synthase O subunit [*Bombyx mori* (domestic silkworm)]	↑ 6.37
XP_973734	myosin-2 essential light chain [*Tribolium castaneum* (red flour beetle)]	↑ 6.34
**Gene ID**	**Gene description [species]**	**CBZ (Fold change)**
XP_681269	hypothetical protein [*Aspergillus nidulans*]	↑ 6.32
XP_790964	copine-8 [*Strongylocentrotus purpuratus* (purple sea urchin)]	↑ 6.26
BAC60471	fadJ multifunctional fatty acid oxidation complex subunit alpha [*Vibrio parahaemolyticus*]	↑ 6.24
ZP_01304133	xylosidase/arabinosidase [*Sphingomonas* sp.]	↑ 6.21
XP_672403	PB300124.00.0 hypothetical protein [*Plasmodium berghei ANKA*]	↑ 6.20
XP_392015	chloride channel protein 2 [*Apis mellifera* (honey bee)]	↑ 6.17
YP_581567	Pcryo_2306 putative DNA helicase [*Psychrobacter cryohalolentis*]	↑ 6.09
XP_725993	PY05524 hypothetical protein [*Plasmodium yoelii yoelii 17XNL*]	↑ 6.05
XP_969486	PREDICTED: similar to Jagged-1 precursor (Jagged1) [Tribolium castaneum]	↑ 6.00
EAT43058	AAEL005474-PA [*Aedes aegypti* (yellow fever mosquito)]	↑ 5.98
BAE02066	uncharacterized [*Macaca fascicularis* (crab-eating macaque)]	↑ 5.87
BAB77866	alr1500 hypothetical protein [*Nostoc* sp.]	↑ 5.85
ABB06938	FAD dependent oxidoreductase [*Burkholderia lata*]	↑ 5.83
CAG09120	unnamed protein product [*Tetraodon nigroviridis*]	↑ 5.80
ABC73068	venom allergen 5 [*Vespula maculifrons*]	↑ 5.78
ABD19264	cytochrome b [*Daphnia pulex*]	↑ 5.76
AAB31526	O-type P element protein {exons 0-3} [*Drosophila bifasciata*]	↑ 5.76
AAC05908	cytochrome oxidase subunit II [*Pieris rapae*]	↑ 5.71
CAB05290	hypothetical protein T27E7.3 T27E7.3 [*Caenorhabditis elegans*]	↑ 5.53
BAD40302	2-oxoacid:ferredoxin oxidoreductase alpha subunit [*Symbiobacterium thermophilum*]	↑ 5.50
CAG07432	unnamed protein product [*Tetraodon nigroviridis*]	↑ 5.49
AAS93718	CG30022 [*Drosophila melanogaster* (fruit fly)]	↑ 5.46
AAH85561	Hypothetical protein [*Danio rerio* (zebrafish)]	↑ 5.41
ZP_01034049	His/Glu/Gln/Arg/opine family ABC transporter, permease protein [*Roseovarius* sp.]	↑ 5.39
EAA08205	AGAP002490-PA [*Anopheles gambiae str. PEST*]	↑ 5.37
XP_394362	E3 ubiquitin-protein ligase UBR1 [*Apis mellifera* (honey bee)]	↑ 5.36
CAB05757	SRX-29 [*Caenorhabditis elegans*]	↑ 5.36
CAA10769	hypothetical protein [*Cryptosporidium parvum*]	↑ 5.34
AAS53791	AFR420Wp [*Ashbya gossypii* ATCC 10895] AFR420Wp [*Eremothecium gossypii*]	↑ 5.11
XP_392616	PREDICTED: similar to ENSANGP00000021560 [*Apis mellifera*]	↑ 5.11
XP_723863	PY00386 CCAAT-box DNA binding protein subunit B [*Plasmodium yoelii yoelii*]	↑ 5.11
XP_636901	DDB_G0288093 RING zinc finger-containing protein [*Dictyostelium discoideum*]	↑ 5.07
CAH03604	PTMB.407 hypothetical protein [*Paramecium tetraurelia strain d4-2*]	↑ 5.04
NP_440321	Sll1510 protein [*Synechocystis*]	↑ 5.03
XP_624692	Glutathione S-transferase T1 [*Apis mellifera* (honey bee)]	↑ 5.02
EAS03884	TTHERM_00455230 hypothetical protein [*Tetrahymena thermophila*]	↑ 5.00
Q25158	Compound eye opsin BCRH2 opsin BcRh2 [*Hemigrapsus sanguineus*]	↑ 4.87
XP_394551	NEDD8-conjugating enzyme UBE2F-like [*Apis mellifera* (honey bee)]	↑ 4.75
EAT45700	AAEL003027-PA [*Aedes aegypti* (yellow fever mosquito)]	↑ 4.74
XP_968298	PREDICTED: similar to CG31543-PC, isoform C [*Tribolium castaneum*]	↑ 4.71
XP_781103	homogentisate 1,2-dioxygenase [*Strongylocentrotus purpuratus* (purple sea urchin)]	↑ 4.64
EAA00702	AGAP011988-PA [*Anopheles gambiae str. PEST*]	↑ 4.57
EAR85282	TTHERM_00470550 phospholipid-translocating P-type ATPase, flippase family protein [*Tetrahymena thermophila*]	↑ 4.57
YP_547797	Bpro_0943 hypothetical protein [*Polaromonas* sp.]	↑ 4.56
AAR33556	cytochrome c oxidase, coo3-type, cytochrome c subunit II, one heme-binding site [*Geobacter sulfurreducens PCA*]	↑ 4.55
NP_194153	3-oxo-Delta(4,5)-steroid 5-beta-reductase. [*Arabidopsis thaliana*]	↑ 4.53
ABA45367	zinc ABC transporter ATP-binding protein [*Streptococcus agalactiae*]	↑ 4.47
ZP_01189085	Excinuclease ABC, A subunit [*Halothermothrix orenii*]	↑ 4.44
EAT43025	AaeL_AAEL005513 AAEL005513-PA [*Aedes aegypti* (yellow fever mosquito)]	↑ 4.44
XP_710938	Potential fungal zinc cluster transcription factor [*Candida albicans*]	↑ 4.40
XP_751922	aminopeptidase [*Aspergillus fumigatus*]	↑ 4.39
AAT74669	cysteine-rich secreted protein 3 [*Mesocestoides vogae*]	↑ 4.39
AAH19729	Usf2 upstream transcription factor 2 [*Mus musculus* (house mouse)]	↑ 4.29
NP_648180	CG13675-PA [*Drosophila melanogaster*]	↑ 4.03

XP_958062	NCU10014 hypothetical protein [*Neurospora crassa*]	↑ 3.92
EAA00530	AGAP012418-PA [*Anopheles gambiae str. PEST*]	↑ 3.89
AAY54998	IP06749p [*Drosophila melanogaster*]	↑ 3.87
BAC24521	minC [*Wigglesworthia glossinidia* endosymbiont of *Glossina brevipalpis*]	↑ 3.86

Gene ID	Gene description [species]	CBZ (Fold change)
XP_001069615	PREDICTED: similar to YY1 transcription factor [*Rattus norvegicus*]	↑ 3.86
BAC12610	hypothetical conserved protein [*Oceanobacillus iheyensis* HTE831]	↑ 3.69
YP_629939	MXAN_1687 hypothetical protein [*Myxococcus xanthus* DK 1622]	↑ 3.53
AAZ06578	pAW63_007 type II intron reverse transcriptase maturase [*Bacillus thuringiensis* serovar kurstaki]	↑ 3.52
YP_621339	Bcen_1460 MscS mechanosensitive ion channel [*Burkholderia cenocepacia*]	↑ 3.48
XP_392121	papilin-like [*Apis mellifera* (honey bee)]	↑ 3.41
AAT89163	hypothetical protein [*Leifsonia xyli* subsp.]	↑ 3.39
XP_763925	TP04_0290 hypothetical protein [*Theileria parva strain Muguga*]	↑ 3.36
AAY66970	secreted protein [*Ixodes scapularis*]	↑ 3.30
XP_695583	similar to HYLS1 protein [*Danio rerio* (zebrafish)]	↑ 3.28
EAA04645	AGAP007365-PA [*Anopheles gambiae str. PEST*]	↑ 3.24
XP_785823	PREDICTED: similar to dispatched homolog 1 [*Strongylocentrotus purpuratus*]	↑ 3.19
XP_521564	Centrosomal protein 55kDa [*Pan troglodytes]*	↑ 3.14
XP_396152	SMSr sphingomyelin synthase-related 1 [*Apis mellifera* (honey bee)]	↑ 3.06
NP_872374	hypothetical protein [Homo sapiens]	↑ 3.03
AAH82673	bcat1 branched chain amino-acid transaminase 1, cytosolic [*Xenopus laevis* (African clawed frog)]	↑ 2.90
BAD46348	Os09g0525400 [*Oryza sativa Japonica Group*]	↑ 2.87
CAG67999	conserved hypothetical protein; putative membrane protein [*Acinetobacter* sp. ADP1]	↑ 2.83
EAL33401	GA18290-PA [Drosophila pseudoobscura]	↑ 2.82
EAT38039	AaeL_AAEL010027 AAEL010027-PA [*Aedes aegypti* (yellow fever mosquito)]	↑ 2.79
YP_293946	EhV192 hypothetical protein [*Emiliania huxleyi virus 86*]	↑ 2.77
EAA04917	AgaP_AGAP000973 AGAP000973-PA [*Anopheles gambiae str. PEST*]	↑ 2.75
EAL27357	gene product from transcript GA20418-RA [*Drosophila pseudoobscura*]	↑ 2.47
ZP_01079163	possible helicase [*Synechococcus* sp.]	↑ 2.47
EAT42364	AAEL006097-PA [*Aedes aegypti* (yellow fever mosquito)]	↑ 2.21
AAT39336	DNA repair protein RAD51 [*Oikopleura dioica*]	↑ 2.20
CAA93496	ALG-1 [*Caenorhabditis elegans*]	↑ 2.17
XP_952900	TA07340 hypothetical protein [*Theileria annulata strain Ankara*]	↑ 2.01
EAR90129	TTHERM_00354760 hypothetical protein [*Tetrahymena thermophila*]	↑ 1.89

Gene ID	Gene description [species]	CBZ (Fold change)
NP_610462	shrb transfer/carrier protein [*Drosophila melanogaster*]	↓ -2.03
EAS00551	hypothetical protein TTHERM_00409040 [*Tetrahymena thermophila*]	↓ -2.03
AAH42230	Ribosomal protein S1a protein [*Xenopus laevis*]	↓ -2.10
XP_458550	hypothetical protein DEHA0D02585g [*Debaryomyces hansenii*]	↓ -2.14
XP_821615	protein kinase [*Trypanosoma cruzi* strain CL Brener]	↓ -2.14
XP_592181	PREDICTED: similar to Y37D8A.2 isoform 1 [*Bos taurus*]	↓ -2.17
XP_686827	PREDICTED: similar to Bmp1 protein [*Danio rerio*]	↓ -2.20
XP_966285	DNA polymerase epsilon catalytic subunit A [*Plasmodium falciparum*]	↓ -2.21
XP_678020	mitochondrial DNA, complete genome [*Triops cancriformis*]	↓ -2.21
XP_541754	guanyl-nucleotide exchange factor [*Canis familiaris*]	↓ -2.27
XP_651053	hypothetical protein 185.t00007 [*Entamoeba histolytica*]	↓ -2.27
NP_079004	succinyl-CoA:glutarate-CoA transferase [*Homo sapiens*]	↓ -2.30
AAH97162	F-box and leucine-rich repeat protein 18 [*Danio rerio*]	↓ -2.33
ZP_00131989	Predicted ATPase [*Haemophilus somnus*]	↓ -2.40
XP_974187	PREDICTED: similar to CG8029-PB, isoform B [*Tribolium castaneum*]	↓ -2.42
NP_766109	nuclear protein in testis [*Mus musculus*]	↓ -2.43
XP_640534	GMP synthetase [*Dictyostelium discoideum*]	↓ -2.44
AAM40505	DNA mismatch repair protein [*Xanthomonas campestris*]	↓ -2.63
BAC10625	ubiquitin conjugating enzyme-like protein [*Bombyx mori*]	↓ -2.64
BAC98829	F-box WD40 protein [*Labidochromis caeruleus*]	↓ -2.65
XP_623472	PREDICTED: similar to ENSANGP00000024947 [*Apis mellifera*]	↓ -2.65
CAI86921	conserved protein of unknown function ; putative membrane protein [*Pseudoalteromonas haloplanktis*]	↓ -2.69
CAE79263	glutaminyl-tRNA synthetase [*Bdellovibrio bacteriovorus*]	↓ -2.71
NP_701577	hypothetical protein PFL1075w [*Plasmodium falciparum*]	↓ -2.71

EAL28674	GA15521-PA [*Drosophila pseudoobscura*]	↓ -2.72
CAE66221	Hypothetical protein CBG11463 [*Caenorhabditis briggsae*]	↓ -2.72
NP_001017150	chromobox homolog 1 [*Xenopus tropicalis*]	↓ -2.77
XP_393841	PREDICTED: similar to AMME syndrome candidate gene 1 protein [*Apis mellifera*]	↓ -2.81
AAT39415	Gaba(A) receptor associated protein [*Branchiostoma belcheri tsingtaunese*]	↓ -2.83
EAT36570	alcohol dehydrogenase [*Aedes aegypti*]	↓ -2.86
XP_624527	repressor of RNA polymerase III transcription MAF1 homolog [*Apis mellifera* (honey bee)]	↓ -2.87

Gene ID	Gene description [species]	CBZ (Fold change)
AAD32568	NT6 [Nicotiana tabacum]	↓ -2.88
XP_859489	PREDICTED: similar to hydroxyacyl dehydrogenase, subunit A isoform 3 [*Canis familiaris*]	↓ -2.91
XP_624997	PREDICTED: similar to oxysterol-binding protein-like protein 9 isoform d [*Apis mellifera*]	↓ -3.00
EAR96527	Major Facilitator Superfamily protein [*Tetrahymena thermophila*]	↓ -3.02
XP_967132	PREDICTED: similar to CG6016-PB, isoform B isoform 1 [*Tribolium castaneum*]	↓ -3.03
NP_851603	microtubule associated serine/threonine kinase 1 [*Rattus norvegicus*]	↓ -3.03
AAK41879	Conserved hypothetical protein [*Sulfolobus solfataricus*]	↓ -3.05
NP_523530	60S ribosomal protein L13 RPL13 [*Drosophila melanogaster*]	↓ -3.12
XP_974675	zinc transporter ZIP1 [*Tribolium castaneum*]	↓ -3.15
NP_701446	hypothetical protein PFL0405w [*Plasmodium falciparum*]	↓ -3.20
XP_361380	hypothetical protein MG03854.4 [*Magnaporthe grisea*]	↓ -3.26
AAH50515	WD repeat domain 8 [*Danio rerio*]	↓ -3.34
XP_362894	hypothetical protein MG08543.4 [*Magnaporthe grisea*]	↓ -3.39
CAA67766	acute phase serum amyloid A (SAA) [*Oncorhynchus mykiss*]	↓ -3.39
AAH77956	MGC80949 protein [*Xenopus laevis*]	↓ -3.41
XP_971851	PREDICTED: similar to NADH-ubiquinone oxidoreductase 42 kDa subunit, mitochondrial precursor (Complex I-42KD) (CI-42KD) [*Tribolium castaneum*]	↓ -3.42
EAT34816	glycine cleavage system h protein [*Aedes aegypti*]	↓ -3.45
CAG31427	annexin A11 [*Gallus gallus*]	↓ -3.46
XP_650833	heat shock protein 70 [*Entamoeba histolytica*]	↓ -3.47
YP_485288	pyrroloquinoline-quinone aldehyde dehydrogenase [*Rhodopseudomonas palustris*]	↓ -3.49
AAV34884	ribosomal protein S27 [*Bombyx mori*]	↓ -3.49
XP_969209	PREDICTED: similar to sphingosine-1-phosphatase [*Tribolium castaneum*]	↓ -3.50
XP_975592	PREDICTED: similar to CG40410-PA.3 [*Tribolium castaneum*]	↓-3.51
XP_221438	PREDICTED: similar to Cdc42 GTPase-activating protein [*Rattus norvegicus*]	↓ -3.51
CAC44629	deafness dystonia protein [*Takifugu rubripes*]	↓ -3.68
XP_974201	39S ribosomal protein L44, mitochondrial [*Tribolium castaneum*]	↓-3.68
EAA13751	AGAP010769-PA glucosamine 6-phosphate N-acetyltransferase [*Anopheles gambiae* str. PEST]	↓ -3.75
EAT40746	conserved hypothetical protein [*Aedes aegypti*]	↓ -3.78
XP_975769	cytochrome b-c1 complex subunit 2, mitochondrial [Tribolium castaneum]	↓ -3.79
XP_001076360	PREDICTED: similar to retinoblastoma binding protein 6 isoform 1 isoform 2 [*Rattus norvegicus*]	↓ -3.97
XP_653493	hypothetical protein 81.t00020 [*Entamoeba histolytica*]	↓ -3.98
XP_971017	zinc transporter ZIP11 [*Tribolium castaneum*]	↓ -4.00
EAL27218	GA18926-PA [*Drosophila pseudoobscura*]	↓ -4.12
ZP_00800575	4Fe-4S ferredoxin, iron-sulfur binding [*Alkaliphilus metalliredigenes*]	↓ -4.16
XP_829758	hypothetical protein Tb11.01.8780 [*Trypanosoma brucei*]	↓ -4.22
XP_726251	hypothetical protein PY00679 [*Plasmodium yoelii yoelii* str.]	↓-4.24
BAD94515	peroxisome proliferator-activated receptor gamma [*Oncorhynchus keta*]	↓ -4.33
CAD67790	No homology	↓ -4.39
BAB05837	hypothetical protein BH2118 [*Bacillus halodurans*]	↓ -4.41
AAT42372	glycogen synthase kinase-3 [*Lytechinus variegatus*]	↓-4.74
XP_001102209	PREDICTED: similar to Methylmalonyl-CoA epimerase, mitochondrial precursor (DL-methylmalonyl-CoA racemase) isoform 1 [*Macaca mulatta*]	↓-4.75
AAC27659	tryptophan oxygenase [*Anopheles gambiae*]	↓ -4.85
NP_039078	ORF FPV115 Ankyrin repeat gene family protein [*Fowlpox* virus]	↓ -4.90
XP_667711	hypothetical protein Chro.70604 [*Cryptosporidium hominis*]	↓ -4.96
ZP_01066446	putative permease [*Vibrio* sp.]	↓ -5.13
XP_696756	PREDICTED: similar to sulfiredoxin 1 homolog [*Danio rerio*]	↓ -5.26
ABD33303	hypothetical protein MtrDRAFT_AC158502g12v1 [*Medicago truncatula*]	↓ -5.32
XP_388815	hypothetical protein FG08639.1 [*Gibberella zeae* PH-1]	↓ -5.47
CAE73165	Hypothetical protein CBG20561 [*Caenorhabditis briggsae*]	↓ -7.21
XP_851407	PREDICTED: similar to serine/cysteine proteinase inhibitor, clade I, member 2 isoform 1 [*Canis familiaris*]	↓ -9.10
AAB01338	EGF repeat transmembrane protein [*Mus musculus*]	↓ -9.69
AAH81106	MGC83377 protein [*Xenopus laevis*]; solute carrier family 5 (sodium/glucose cotransporter),	↓ -12.11

	member 2	
EAT39824	inorganic-stress: Fullerene nanoparticle, normal: Females-adult [*Aedes aegypti*]	↓ -17.08
XP_420864	T-cell surface glycoprotein CD8 alpha chain-like; PREDICTED: similar to CD8 alpha chain precursor [*Gallus gallus*]	↓ -24.06
AAP77784	hypothetical protein HH_1187 [*Helicobacter hepaticus*]	↓ -7396.75
XP_700169	PREDICTED: similar to conserved hypothetical protein [*Danio rerio*]	↓ -10187.81
AAR01249	laccase 8 [*Coprinopsis cinerea*]	↓ -10930.12
AAH00967	NudC domain containing 1 [*Homo sapiens*]	↓ -11402.40
XP_500810	SEC16, ISOFORM F; hypothetical protein [*Yarrowia lipolytica*]	↓ -13979.40
AAQ75727	NADH dehydrogenase I [*Errhomus variabilis*]	↓ -20664.18

Gene ID	Gene description [species]	CBZ (fold change)
XP_784306	PREDICTED: similar to placental protein 11 related [*Strongylocentrotus purpuratus*]	↓ -29883.84
ZP_01181739	Phage minor structural protein, N-terminal [*Bacillus cereus* subsp.]	↓ -35745.20
XP_765680	hypothetical protein TP01_0153 [*Theileria parva* strain Muguga]	↓ -59189.85
AAM51523	Hypothetical protein C28G1.6 [*Caenorhabditis elegans*]	↓ -69117.57
ZP_00851049	hypothetical protein Shewana3DRAFT_1897 [*Shewanella* sp.]	↓ -98465.68
XP_385040	hypothetical protein FG04864.1 [*Gibberella zeae*]	↓ -109521.08
XP_973845	apoptosis-resistant E3 ubiquitin protein ligase 1 [*Tribolium castaneum*]	↓ -116578.52

b) F12 generation

Gene ID	Gene description [species]	CBZ (fold change)
EAA04403	AGAP006931-PA; ENSANGP00000021782 [*Anopheles gambiae* str. PEST]	↑ 72368.51
YP_548045	hypothetical protein Bpro_1196 [*Polaromonas* sp.]	↑ 71962.22
EAL26781	GA20714-PA [*Drosophila pseudoobscura*]	↑ 56738.63
CAD67790	double stranded RNA-activated protein kinase 1 [*Tetraodon nigroviridis*]	↑ 35149.50
XP_827078	hypothetical protein Tb09.160.5290 [*Trypanosoma brucei*]	↑ 28898.11
XP_665805	hypothetical protein Chro.60399 [*Cryptosporidium hominis*]	↑ 22473.06
AAM74161	Pax-6 protein [*Euprymna scolopes*]	↑ 18679.08
YP_476405	ABC1 domain protein [*Synechococcus* sp.]	↑ 18288.22
ABG52453	peptidase M23B [*Trichodesmium erythraeum*]	↑ 17199.64
BAE56654	unnamed protein product [Aspergillus oryzae]	↑ 17137.55
CAG76660	conserved hypothetical protein [*Erwinia carotovora* subsp. atroseptica]	↑ 16193.51
NP_008814	NADH dehydrogenase subunit 6 [*Mustelus manazo*]	↑ 15117.52
XP_394766	PREDICTED: similar to CG33175-PG, isoform G, partial [*Apis mellifera*]	↑ 14893.60
AAH88813	lectin, galactoside-binding, soluble, 9B;; Lgals9-prov protein [*Xenopus tropicalis*]	↑ 14143.80
EAR91277	transmembrane protein, putative; hypothetical protein TTHERM_00784640 [*Tetrahymena thermophila*]	↑10882.44
NP_922637	hypothetical protein [*Oryza sativa* (japonica cultivar-group)]	↑ 10539.98
XP_765680	hypothetical protein TP01_0153 [*Theileria parva* strain Muguga]	↑ 10199.45
NP_492661	MUTator family member (mut-16) [*Caenorhabditis elegans*]	↑ 8548.91
AAT64428	pMGA 1.4 [*Mycoplasma gallisepticum*] haemagglutinin homologue	↑ 8363.90
CAD51508	asparagine--tRNA ligase, putative [*Plasmodium falciparum*]	↑ 7605.56
ZP_00637578	hypothetical protein SfriDRAFT_3544 [*Shewanella frigidimarina*]	↑ 5909.83
ABB44488	Suden_1210 hypothetical protein [*Sulfurimonas denitrificans*]	↑ 4916.25
BAD94515	peroxisome proliferator-activated receptor gamma [*Oncorhynchus keta*]	↑ 4835.57
AAY54998	IP06749p [*Drosophila melanogaster*]	↑ 3815.65
AAH74846	transmembrane protease, serine 3; Transmembrane protease, serine 3, isoform 1 [*Homo sapiens*]	↑ 3770.56
XP_465194	putative speckle-type POZ protein [*Oryza sativa* (japonica cultivar-group)]	↑ 3072.81
P05842	Putative noncapsid protein NS-1 (Nonstructural protein NS1) (NCVP1)	↑ 2264.67
XP_001091323	solute carrier family 15, member 5 [*Macaca mulatta*]	↑ 10.76
XP_785823	PREDICTED: similar to dispatched homolog 1 [*Strongylocentrotus purpuratus*]	↑ 9.43
BAD74252	type IIs restriction endonuclease; type IIs restriction endonuclease [*Geobacillus kaustophilus*]	↑ 7.78
XP_430000	PREDICTED: hypothetical protein [*Gallus gallus*]	↑ 7.35
XP_981685	PREDICTED: hypothetical protein [*Mus musculus*]	↑ 6.23
XP_001069615	PREDICTED: similar to YY1 transcription factor [*Rattus norvegicus*]	↑ 6.15
ZP_01001041	LacI family regulatory protein [*Oceanicola batsensis*]	↑ 5.16
AAY66970	secreted protein [*Ixodes scapularis*]	↑ 3.37

XP_001063788	Keratin associated protein 20-like 2 [*Rattus norvegicus* (Norway rat)]	↑ 3.29
AAH70339	Deoxyuridine triphosphatase DUT [*Homo sapiens*]	↑ 3.21
CAC95124	TIR/NBS/LRR protein [*Populus deltoides*]	↑ 3.09
EAT41358	AAEL007022-PA [*Aedes aegypti*(yellow fever mosquito)]	↑ 2.80
AAK52091	Cth cystathionine gamma-lyase [*Rattus norvegicus* (Norway rat)]	↑ 2.74
XP_746198	PC001062.02.0 hypothetical protein [*Plasmodium chabaudi chabaudii*]	↑ 2.71
T44130	hypothetical protein [imported] - [*Staphylococcus aureus*] (fragment)	↑ 2.63
XP_813347	UDP-glucoronosyl and UDP-glucosyl transferase [*Trypanosoma cruzi* strain CL Brener]	↑ 2.42
XP_541754	guanyl-nucleotide exchange factor [*Canis familiaris*]	↑ 2.40
XP_969056	ubiquitin carboxyl-terminal hydrolase 14 [*Tribolium castaneum* (red flour beetle)]	↑ 2.40
ZP_01376161	hypothetical protein Ccur5_01001129 [*Campylobacter curvus*]	↑ 2.29
XP_974201	PREDICTED: 39S ribosomal protein L44, mitochondrial [*Tribolium castaneum*]	↑ 2.25
Gene ID	Gene description [species]	CBZ (Fold change)
AAZ75599	CRISP-ENH2 [*Pseudoferania polylepis*]	↑ 2.13
XP_971017	PREDICTED: zinc transporter ZIP11 isoform X2 [*Tribolium castaneum*]	↑ 2.08
AAH74592	MGC69530 protein [*Xenopus (Silurana) tropicalis*]	↑ 2.01
P34724	RecName: Full=Acid phosphatase; Flags: Precursor [*Aspergillus niger*]	↑ 2.00
XP_393267	PREDICTED: testican-1-like [*Apis mellifera*]	↑ 1.95
XP_966285	DNA polymerase epsilon catalytic subunit A [*Plasmodium falciparum*]	↑ 1.72

Gene ID	Gene description [species]	CBZ (Fold change)
CAE73165	Hypothetical protein CBG20561 [*Caenorhabditis briggsae*]	↓ -2.61
XP_651175	chloride channel protein 2[*Entamoeba histolytica*]	↓ -2.91
EAA08389	AGAP003192-PA [*Anopheles gambiae* str. PEST]	↓ -3.05
ABE93099	hypothetical protein MtrDRAFT_AC122172g1v2 [*Medicago truncatula*]	↓ -3.44
CAF89999	unnamed protein product [*Tetraodon nigroviridis*]	↓ -3.73
NP_440283	histidinol dehydrogenase [*Synechocystis* sp]	↓ -3.76
I30010	SMU_1069c hypothetical protein [*Streptococcus mutans* UA159]	↓ -3.77
EAT43025	mothers against dpp protein [*Aedes aegypti* (yellow fever mosquito)]	↓ -3.90
XP_581858	PREDICTED: similar to Valyl-tRNA synthetase (Valine--tRNA ligase) (ValRS) (G7a protein) [*Bos taurus*]	↓ -3.92
XP_975669	PREDICTED: hypothetical protein [*Tribolium castaneum*]	↓ -3.97
XP_973543	polypeptide N-acetylgalactosaminyltransferase 5 [*Tribolium castaneum* (red flour beetle)]	↓ -3.97
EAT34457	conserved hypothetical protein [*Aedes aegypti*]	↓ -4.01
XP_678020	hypothetical protein [*Plasmodium berghei* ANKA]	↓ -4.07
XP_710938	Potential fungal zinc cluster transcription factorspecies: Candida albicans/putative transcription factor [*Candida albicans*]	↓ -4.12
XP_640018	hypothetical protein [*Dictyostelium discoideum* AX4]	↓ -4.13
YP_547797	hypothetical protein Bpro_0943 [*Polaromonas* sp.]	↓ -4.38
ABB06938	FAD dependent oxidoreductase [*Burkholderia lata*]	↓ -4.42
NP_571006	solute carrier family 39 (zinc transporter), member 7 [*Danio rerio* (zebrafish)]	↓ -4.56
XP_956378	hypothetical protein [*Neurospora crassa* OR74A]	↓ -4.64
EAA08205	AGAP002490-PA [*Anopheles gambiae* str. PEST]	↓ -4.67
AAA29908		
